# New insights on familial colorectal cancer type X syndrome

**DOI:** 10.1038/s41598-022-06782-8

**Published:** 2022-02-18

**Authors:** Felipe Antonio de Oliveira Garcia, Edilene Santos de Andrade, Henrique de Campos Reis Galvão, Cristina da Silva Sábato, Natália Campacci, Andre Escremin de Paula, Adriane Feijó Evangelista, Iara Viana Vidigal Santana, Matias Eliseo Melendez, Rui Manuel Reis, Edenir Inez Palmero

**Affiliations:** 1 Molecular Oncology Research Center, Barretos Cancer Hospital, Antenor Duarte Villela Street, 1331, Barretos, São Paulo CEP 14784-400 Brazil; 2 Oncogenetics Department, Barretos Cancer Hospital, Barretos, São Paulo Brazil; 3 Center of Molecular Diagnosis, Barretos Cancer Hospital, Barretos, São Paulo Brazil; 4 Pathology Department, Barretos Cancer Hospital, Barretos, São Paulo Brazil; 5 Department of Molecular Carcinogenesis, Brazilian National Cancer Institute, Rio de Janeiro, Brazil; 6 Life and Health Sciences Research Institute (ICVS), Medical School, University of Minho, Braga, Portugal; 7 ICVS/3B’s-PT Government Associate Laboratory, Braga/Guimarães, Portugal; 8 Department of Genetics, Brazilian National Cancer Institute, Rio de Janeiro, Brazil

**Keywords:** Cancer, Genetics, Molecular biology, Molecular medicine, Oncology

## Abstract

Familial colorectal cancer type X (FCCTX) is a heterogeneous colorectal cancer predisposition syndrome that, although displays a cancer pattern similar to Lynch syndrome, is mismatch repair proficient and does not exhibit microsatellite instability. Besides, its genetic etiology remains to be elucidated. In this study we performed germline exome sequencing of 39 cancer-affected patients from 34 families at risk for FCCTX. Variant classification followed the American College of Medical Genetics and Genomics (ACMG) guidelines. Pathogenic/likely pathogenic variants were identified in 17.65% of the families. Rare and potentially pathogenic alterations were identified in known hereditary cancer genes (*CHEK2*), in putative FCCTX candidate genes (*OGG1* and *FAN1)* and in other cancer-related genes such as *ATR, ASXL1, PARK2, SLX4* and *TREX1.* This study provides novel important clues that can contribute to the understanding of FCCTX genetic basis.

## Introduction

Hereditary cancer accounts for approximately 20–30% of colorectal cancer (CRC) cases^1^. Lynch syndrome (LS) is one of the main CRC predisposition syndromes. Familial colorectal cancer type X (FCCTX) can be considered a subgroup of LS. Both are quite similar in their clinical presentation and characterized by several early onset nonpolyposis hereditary colorectal cancer cases in patients and family members from different generations^2^. From a clinical perspective, both the FCCTX and Lynch families are characterized by the fulfilment of Amsterdam I clinical criteria^3^. However, FCCTX and LS differ at the molecular level, because LS is caused by mismatch repair genes (MMR) *MLH1, MSH2, MSH6,* and *PMS2* malfunctioning, thus presenting microsatellite instability (MSI—high—high microsatellite instability). Conversely, FCCTX patients do not harbour any alteration in the MMR genes and, as a consequence, carry no alterations in microsatellites, being microsatellite stable (MSS)^4^. FCCTX tumours are also heterogeneous, occurring mainly as moderately differentiated adenocarcinomas in the rectum and sigmoid regions of the large intestine^5^. Cancer-related death is up to 10 years higher in FCCTX than in Lynch syndrome patients. Additionally, although the risk for developing a second CRC is higher for Lynch, the risk with FCCTX shows a higher proportional increase. Besides, patients with FCCTX have tumours with diagnosis in advanced ages than in Lynch and have fewer extracolonic tumours^6^.

The understanding of FCCTX molecular mechanisms is poorly explored. Some genes have already been reported to be potentially associated with FCCTX, namely *BMPR1A*^7^*, RPS20*^8^*, SEMA4A*^9^*, SETD6*^10^*, BRCA2*^11^*, OGG1*^12^ and *FAN1*^13^*.* Besides, a review study suggested a possible association with *CENPE, CHD18, GREM1, BCR, KIF24, GALNT12, ZNF367, HABP4, GABBR2,* and *BMP4*^14^. In addition, a review by Nejadtagui and collaborators^15^ pointed *BRCA2*, *KRAS*, *APC*, *MGMT*, *BRAF*, *BMPR1A*, *RPS20*, *SEMA4A,* and hypermethylation of at least one gene of the MMR system as potentially related to FCCTX. Despite these studies, no defined set of genes is conclusively associated with FCCTX. Therefore, we propose to perform a clinical and molecular characterization of a cohort of families fulfilling the clinical criteria for FCCTX syndrome.

## Results

### Clinical pathological information

The socio-demographic and histopathological data are summarized in Table 1. Twenty-eight patients were female (71.8%), with an average age of 49 years at diagnosis. Most of the primary tumours were colorectal (92.3%) and these ones were mostly located in the rectum (35.9%). Besides, three patients with colorectal cancer had other extra-colonic primary tumours (endometrium, prostate, and renal).

**Table 1:** Socio-demographic and histopathological features.

	Amount (%)
**Sex**	
Female	28 (71.8)
Male	11 (28.2)
**Ethnicity (self-declared)**	
White	29 (74.3)
Brown	5 (12.8)
Black	3 (7.6)
Yellow	1 (2.5)
Unavailable	1 (2.5)
Diagnostic age (average; minimum and maximum)	Years of age 49.4 (34–77; SD: 11.3)
**Histology**	
Adenocarcinoma (NOS*)	37 (94.9)
Unavailable	2 (5.1)
Primary tumour location**	
Colorectal	36 (92.3)
Endometrium	3 (7.7)
**Location (colorectal)**	
Rectum	14 (38.8)
Sigmoid	7 (19.4)
Right colon	7 (19.4)
Left colon	4 (11.1)
Transversal colon	2 (5.5)
Unavailable	2 (5.5)
**CRC differentiation level**	
Poorly differentiated	9 (25)
Moderately differentiated	24 (66.6)
Well differentiated	0
Unavailable	3 (8.3)
**CRC TNM stating**	
I	8 (22.2)
II (A, B, C)	10 (27.7)
III (A, B, C)	13 (36.1)
IV (A, B)	2 (5.5)
Unavailable	3 (8.3)
**Status**	
Follow up (following with no disease)	28 (71.7)
Deceased	7 (18)
Alive in treatment	1 (2.5)
Loss of follow up	3 (7.7)

*Not otherwise specified.

**Three patients with colorectal cancer also had prostate, endometrium and renal tumors as second primary tumors.

### Family history information

Details about the cancer history of the probands and their relatives are provided in Table 2. For all of the families analysed (n = 34), 22 fulfilled Amsterdam 1 criteria and 10 were quasi-Amsterdam, fulfilling three among the four required criteria (what we called for the purpose of the study as “Amsterdam-1” (minus one)), and two families fulfilled two out of the four criteria (here called “Amsterdam-2”). For three families, two relatives were analysed (ID 17, 23, 26), and for one family, three relatives were included (ID 21).

**Table 2:** Family history.

Family ID*	Criteria fulfilled	Index case cancer (sex, age at diagnosis)	Cancer family history Cancer type (sex, age at diagnosis)
1	Amsterdam	Colorectal M41	Colorectal (M36, M34, F50, M?, M?), Hepatic (M49), Uterine (F50, F45), Urothelial (M?)
2	Amsterdam	Colorectal M38	Colorectal (F40, F40)
3	Amsterdam-1	Colorectal M35	Colorectal (M68)
4	Amsterdam	Colorectal F41	Colorectal (F45, M55, M40), Lung (M70, M?)
5	Amsterdam-1	Colorectal F38	Colorectal (M70), Breast (F50, F52)
6	Amsterdam-1	Colorectal M54	Colorectal (F42), Digestive (M50, M50, F42)
7	Amsterdam	Colorectal F46	Colorectal (M68, M60)
8	Amsterdam	Colorectal F40	Colorectal (M49, F?, M50, F69, M?), Gastric (F65), Uterine (F28)
9	Amsterdam	Colorectal M62	Colorectal (M55, M67), Lung (M?), Sarcoma (M?), Bladder (M?), Central Nervous System (M?), Lymphoma (M?)
10	Amsterdam	Colorectal F34	Colorectal (F28, M60), Polyps (F23), Ovary (F55), Bladder (M80)
11	Amsterdam	Colorectal F67	Colorectal (M70, F62, F40), Oropharynx (M45)
12	Amsterdam	Endometrium F52	Colorectal (M55, M30), “Cancer” (M?), Dysembryoplastic neuroepithelial tumour (M?), Langerhans cell histiocytosis (M?), Kidney (M50), Leukaemia (M68), Thyroid (F35, F35)
13	Amsterdam	Colorectal F37	Colorectal (M54, F70), Central Nervous System (M45), Gastric (F60)
14	Amsterdam	Colorectal M54	Colorectal (M43, F70), Skin (F55, M90)
15	Amsterdam-1	Endometrium F57	Colorectal (M54, F53, M74), Gastric (M?), Mouth (M?)
16	Amsterdam	Colorectal F74	Colorectal (M70, M70, M81, F51, F?, F40), Renal (M30), Bladder (M81), Lung (M82), Skin (F74), Ovary (F?)
18	Amsterdam	Colorectal M56	Colorectal (F30, F30), Kidney (M56), Breast (F63), “Cancer” (M16), Central Nervous System (M30, M42), maxillary sinus (F?)
19	Amsterdam	Colorectal F51	Colorectal (M68, M32), Hodgkin Lymphoma (F55), “Cancer” (M58), Kidney (F66), Leukaemia (F7), Meningioma (F7)
20	Amsterdam-1	Colorectal 68	Colorectal (M90), Breast (F?), “Cancer” (F50), Thyroid (F25), Oesophageal (F60),
22	Amsterdam-1	Colorectal F77	Colorectal (F38), Lung (F47), Breast (F50)
24	Amsterdam	Colorectal M70	Colorectal (M66, F45), Prostate (M70, M?)
25	Amsterdam-1	Colorectal F55	Colorectal (M50), Polyps (M?), Gastric (M52, F60), Epidermoid carcinoma (F55), Breast (F?, F38), Skin (F78, F?), “Cancer” (M60, M60), Prostate (M?), Uterine (F65)
27	Amsterdam	Colorectal F67	Colorectal (F31, F78), Osteosarcoma (M?)
28	Amsterdam	Colorectal F46	Colorectal (M24, F60)
29	Amsterdam-1	Colorectal F41	Liver (M51), Gastric (F?, F61, F?)
30	Amsterdam	Colorectal F40	Colorectal (M58, M82)
31	Amsterdam	Colorectal F29	Colorectal (M80, F42), Prostate (M80), Throat (M70, F?), Gastric (F?), Villous tubular adenoma (F?)
32	Amsterdam	Colorectal F37	Colorectal (M50, F50), Polyps (F?), “Cancer” (M50)
33	Amsterdam	Colorectal M37	Colorectal (F60, F?, M?), Basocellular (F60, F?), Breast (F50), Nasal (F?)
34	Amsterdam-2	Colorectal M58	Colorectal (M58, M60), Lymphoma (M80), Stomach (M80)
**Families with multiple members analyzed**			
17	Amsterdam-1	Colorectal M45	**Colorectal (M51 (17.1)),** Bladder (M45), Polyps (F?, F?), “Cancer” (F?, F?, F?)
21	Amsterdam	Colorectal M49	**Colorectal (M50, M72 (21.1), F49 (21.2)),** Cerebral (M65, M58), Breast (F?), Polyps (F?), Multiple Myeloma (F?), “Cancer” (F?, F?, F?)
23	Amsterdam-1	Endometrium F58	**Colorectal (M33 (23.1)**, F?), Polyps (M?, M?, M?, F?), Myelodysplastic syndrome (M?), Breast (F70, F45, F50), Thyroid (F50), Melanoma (F50)
26	Amsterdam-2	Colorectal F42	**Colorectal (F51 (26.1)),** Endometrial (F47), Cervical (F42), “Cancer” (M?, M?)

*Patient ID follows the family ID. Families with more than one individual analysed have a second number separated by a dot (.) (highlighted in bold); M = Male, F = Female; ? = Unknown age at diagnosis.

The families presented an average of six members with cancer. CRC was the most common type of cancer, with 76 cases in 33 families, median of 2.3 per family, diagnosed at an average age of 56.42 years old. CRC was followed by breast, gastric/digestive, and uterine tumours (uterus, endometrium, and cervix).

### Molecular data

#### Germline variants

We identified 842 variants that passed the in silico filters (Fig. 2) and, following manual prioritization, 514 variants were eliminated for being considered benign or likely benign (ACMG class I or II), sequencing artefacts, or hypermutated gene regions. Of the 323 remaining variants, eight were classified as likely pathogenic or pathogenic. The remaining 315 variants were considered VUS (or class III) for lack of benign/pathogenic evidence. From these VUS, 70 were located in genes related to hereditary cancer, and 42 were in DNA repair genes (Supplementary Tables 2–6).

### Likely pathogenic and pathogenic variants

Of the eight candidate variants (identified in six patients), three were classified as pathogenic (class V ACMG) and five as likely pathogenic (class IV ACMG). Three were missense, one frameshift insertion, three frameshift deletions, and one nonsense variant. The variant information is shown in Table 3, while the patient information is detailed in Table 4. The pedigrees are displayed in Supplementary Figs. 5 to 10.

**Table 3:** Likely pathogenic and pathogenic variants information.

Family ID	Gene	c	p	ClinVar*	Revel	M-CAP	ACMG	Function (hereditary syndrome)***
3	*ATR*	c.3043C > T	p.Arg1015Ter	NF	NA	NA	V	FA (Familial cutaneous telangiectasia and cancer syndrome)
5	*PARK2*	c.758G > A	p.Arg253His	NF	0.752	0.203989	IV	TSG
5	*SLX4*	c.4259dupC	p.Pro1420fs	NF	NA	NA	IV	FA and HR
6	*OGG1*^*#*^	c.30dupC	p.Arg10fs	NF	NA	NA	IV	BER
13	*TREX1*	c.506G > A	p.Arg169His	Pathogenic**	0.828	0.376515	V	MMR
22	*ASXL1*	c.1927dupG	p.Gly642fs	Pathogenic	NA	NA	V	Chromatin regulation (leukaemia)
33	*FAN1*	c.356_357del	p.Arg119fs	NF	NA	NA	IV	FA (Hereditary colorectal cancer)
33	*CHEK2*	c.470T > C	p.Ile157Thr	Pathogenic**	0.538	NA	IV	Cell cycle regulation (Breast and colorectal cancer)

NF = not found; NA = not available; *Consulted at 12/2019, 01/2020, 02/2020; **Although the official status is conflicting, we considered the pathogenic studies. ***Information from Das et al^17^. and GeneCard^19^. FA = Fanconi anaemia pathway, TSG = tumour suppressor gene, HR = homologous recombination, BER = base excision repair, MMR = mismatch repair; ^#^alteration found in homozygosis.

**Table 4:** Patient (harbouring class IV and V variants) information.

Family	Criteria fulfilled	Proband tumour	Diagnostic age (in years)	Germline variant	Tumour location	Tumour differentiation
3	Amsterdam-2	CRC	34	*ATR*:c.3043C > T	Rectum	Moderate
5	Amsterdam-2	CRC	38	*PARK2:c.758G* > *A*	Left colon	Moderate
5	Amsterdam-2	CRC	38	SLX4:c.4259dupC	Left colon	Moderate
6	Amsterdam-2	CRC	54	*OGG1*:c.30dupC	Rectum	Poor
13	Amsterdam-1	CRC	37	*TREX1*:c.506G > A	Rectum	Moderate
22	Amsterdam-2	CRC	77	ASXL1:c.1927dupG	Rectum	Moderate
33	Amsterdam-1	CRC	37	*CHEK2*:c.470T > C	Right colon	Moderate
33	Amsterdam-1	CRC	37	FAN1:c.356_357del	Right colon	Moderate

One of the potentially pathogenic variants found was the missense c.470T > C (p.Ile157Thr) on *CHEK2* gene, a gene known for its association with CRC and breast cancer^16^. This patient (ID 33) also had a likely pathogenic frameshift variant in the *FAN1* gene (c.356_357del; p.Arg119fs), a gene involved in the Fanconi anaemia pathway^13,17^ that interacts with MMR genes/proteins^18^. The patient had moderately different adenocarcinomas in the right colon. On the maternal side of the family, CRC and breast cancer cases have been reported (Supplementary Fig. 5).

A homozygous frameshift likely pathogenic alteration was found in the *OGG1* gene (c.30dupC; p.Arg10fs) (ID6; Supplementary Fig. 6). *OGG1* belongs to the base excision repair pathway^17^, and its protein is an enzyme (8-oxoguanine) that works repairing oxygen reactive DNA lesions (Source: MedlinePlus, National Library of Medicine). The frameshift insertion was located at the beginning of the gene upstream of the two protein domains, reinforcing its pathogenic effect. The patient (ID 6) was diagnosed at 54 years old with poorly differentiated adenocarcinoma located in the rectum and died at 60 years old. During the interview, the patient stated that a cousin and two uncles from the father’s side were diagnosed with gastric cancer, 14 deceased uncles also from the father’s side had “stomach problems”. Besides, his sister had a diagnosis of CRC at 42 years old.

Another frameshift variant identified is located in the *ASLX1* gene (c.1927dupG; p.Gly642fs). The patient (ID 22) was diagnosed at 77 years old with moderately differentiated adenocarcinoma in the rectum. He had one daughter diagnosed with CRC at 38 years old (Supplementary Fig. 7). According to STRING^18^, ASXL1 works with several transcription factors and cell cycle regulators.

Two likely pathogenic variants were identified in patient ID 5: the missense variant c.758G > A (Arg253His) in the gene *PARK2* (or PRKN2) and the frameshift insertion c.4259dupC (p.Pro1420fs) in the DNA repair gene *SLX4*. The patient was diagnosed at 38 years old with moderately differenced adenocarcinoma in the left colon (Supplementary Fig. 8). SLX4 works with several DNA repair pathways, nonhomologous end joining, homologous recombination, Fanconi anaemia, nucleotide excision repair, and some nonspecific pathways^18^.

A nonsense class V alteration in the *ATR* gene was identified in patient ID 3. The patient was diagnosed at 34 years old with moderately differenced adenocarcinoma in the rectum, and his father died with CRC (Supplementary Fig. 9). The *ATR* gene has an important role in several repair pathways and acts as a checkpoint activator during the cell cycle^17,19,20^. The c.3043C > T (p.Arg1015Ter) nonsense variant is located before all three protein domains^21,22^.

A pathogenic missense variant was identified in a patient with colorectal cancer at 37 years of age (ID 13). The c.506G > A (pArg169His) variant was identified in the *TREX1* gene. The patient was diagnosed with moderately differenced adenocarcinoma in the rectum. Her family had two more CRC cases (Supplementary Fig. 10). The key function of *TREX1* is digesting (degrade, metabolize) cytosolic ssDNA, which is stemmed from endogenous retroelements or abnormal replication intermediates, to suppress cell-intrinsic initiation of autoimmunity^23^. Besides, *TREX1* encodes a repair exonuclease that acts on terminal mismatched regions^17,19^ .

### Variants of unknown significance (VUS)

We identified three VUS in unrelated patients (ClinVar and by ACMG criteria) in the MMR genes (Table 5). All tumours had a normal IHC result and were MSS.

**Table 5:** Variants in lynch syndrome genes.

ID	Gene	c	p	Revel	M-CAP	ClinVar*	ACMG
23	*MLH1*	c.2027T > C	p.Leu676Pro	0.917	0.473622	VUS	III
30	*MSH6*	c.2177T > A	p.Phe726Tyr	0.678	0.190663	VUS	III
34	*MSH2*	c.2785C > T	p.Arg929Ter	*NA*	*NA*	VUS	III

*Consult in 03/2021.

*NA* not available.

In the four families with more than one person evaluated (families IDs 17, 21, 23, 26), we did not identify any likely pathogenic or pathogenic variant in these four families. Nonetheless, we identified 26 variants of unknown significance that segregated in every participating member: eight in family 17, seven in family 21, three in family 24 and eight in family 26. Beyond the likely pathogenic *FAN1* variant identified in family ID33, we detected two more variants in the gene that segregated in family 24 and another in family 26. Despite the arising evidence showing a possible relationship between *FAN1* and colorectal cancer or even with FCCTX, we had not enough evidence to categorize the variants in any pathogenic class. Nevertheless, a study shows that the variant FAN1:Met50Arg (which have a deleterious prediction by REVEL and was identified segregating in affected relatives from two of our families) impacts the repairing system causing genetic instability, possibly representing a cancer risk factor^24^. Although, according to the authors, it is not clear if the gene malfunctioning itself is enough to start the carcinogenesis. Another gene related to colorectal cancer segregating (in one family) was *MSH5*, but, despite having a deleterious score and segregating, the patients had no microsatellites instability and adding the lack of scientific information available for this variant, it remained with unknown significance. The pedigrees are displayed in Supplementary Figs. 1 to 4 and a detailed list of the variants in Table S6.

For most of the VUS identified, there is still insufficient evidence available to allow their classification. Sixty-five were located in genes related to hereditary syndromes, being *FAN1, RASAL1, SDHA, ERCC2,* and *TRIM28* the genes with the higher number of VUS identified. Another 44 VUS were in repair pathway genes such as *POLG, ATM, ERCC2, MSH6, PARP3,* and *POLL*. The last group, the carcinogenesis related genes, contained 232 VUS, and the top mutated were *TBP, POLG, BIRC6, EPHA8, MIB2 and WNK4.*

Variants of unknown significance for several genes involved in DNA repair pathways were also identified in more than one family. Among them, we highlight POLG (with variants in eight unrelated families), POLE and POLH (with VUS in four unrelated families each), ATM (with variants in seven families). Moreover, FAN1, XPC and PARP3 genes harboured VUS in four unrelated families (for more details about the variants, see tables S2 to S4).

Variants of unknown significance identified can be found in Supplementary Tables 2 to 6.

## Discussion

In this study, among the 34 families evaluated, we found that six of them (17.65%) presented a potentially pathogenic variant. Most of the genes were involved in DNA damage repair. One of them is the known CRC predisposing gene *CHEK2*^16,25,26^. In addition, pathogenic/likely pathogenic variants were found in genes previously associated with FCCTX/hereditary CRC as *OGG1*^12,27^ and *FAN1*^13^. Furthermore, potentially pathogenic variants were identified in the *ATR*, *TREX1, ASXL1, PARK2* and *SLX4* genes. Although likely pathogenic and pathogenic variants were confirmed by Sanger sequencing, the association of the variants identified with the FCCTX family history requires further validation by segregation analysis, and in other familial cancer cohorts. Moreover, specific variants would also require in vitro analyses to investigate functional consequences for protein function.

Among the genes where a potentially pathogenic variant was identified, one of them was already associated with hereditary colorectal cancer, the *CHEK2* gene^16,26^. The identified variant (c.470T > C) has already been associated with hereditary and sporadic CRC^25^. Suchy et al. identified this variant in Amsterdam I families negative for MMR alterations and concluded that it confers risk for hereditary CRC^26^. Liu et al. published a meta-analysis with 4029 cases and 13,844 controls and showed that the variant confers a moderate risk for hereditary CRC (odds ratio = 1.97, 95% CI 1.41–2.74, P < 0.001)^25^.

The *CHEK2* mutated patient is also carrier of a frameshift deletion at *FAN1*, whose association with FCCTX has been described previously^13^. Segui and collaborators performed Exome sequencing of three family members (Amsterdam I and MSS) and 176 other families with a history of CRC and concluded that the malfunctioning of Fanconi anaemia pathway (due to *FAN1* alteration) might predispose patients to CRC^13^. *FAN1* works to support the MMR system^18^, which is already classically associated with hereditary CRC^1^. In addition, our group reported a missense *FAN1* variant was identified in two families, one with a strong history of breast and CRC cases^28^. Considering the fact that both, *CHEK2* and *FAN1* can lead to a low to moderated increase in the CRC risk, we believe that a model with an additive effect of both variants can be feasible. This hypothesis should be further evaluated through segregation assays as well as with functional assays.

Other four families with loss of function pathogenic/likely pathogenic variants were identified. Among the frameshift alterations, c.30dupC in the *OGG1* gene was identified in homozygosis, in a family where CRC cases were present in the proband and her sister. Besides, 19 other relatives with stomach problems/ “digestive” tumours were reported. Published studies have shown that missense alterations in *OGG1* may confer a risk for early onset CRC^12,29^. Garre and collaborators evaluated 42 MSS-HNPCC families and identified a missense alteration in *OGG1* that affects splicing. The alteration was detected in an Amsterdam I family and co-segregated with cancer^27^. A meta-analysis from Zhange & Mo with 5235 cases and 8438 controls also concluded that polymorphisms in *OGG1* confer risk for CRC, especially in Caucasians^30^. Concerning the relation between *OGG1* and gastric cancer, two case–control studies showed that polymorphisms in *OGG1* confer risk for gastric cancer, as did a meta-analysis with 1180 cases, and 2444 controls^31–33^.

The other cancer susceptibility gene for which a potentially pathogenic frameshift variant was found is *ASLX1*, a classic leukaemia-associated gene related to Bohring-Optiz syndrome, which increases the risk for Wilms tumours^34,35^. To the best of our knowledge, there have been no previous associations with CRC. In the family carrying the *ASLX1* pathogenic variant, no leukaemia or myelodysplasia cases have been reported, reinforcing the need for further studies evaluating the association of these genes with the phenotypes observed in the family.

One family carrying two likely pathogenic variants (at *SLX4* and *PARK2* genes) was identified in our cohort. Frameshift variants at *SLX4* gene have already been detected in sporadic CRC^36^, and the gene is known for its association with hereditary breast and gastric cancer^37^. Regarding the *PARK2* gene, it has no clear function to date. Ikeuchi et al. detected tumour suppressor activity by negatively regulating the cell cycle in colorectal cancer cell lines^38^. Pologiannis et al. detected *PARK2* copy number alterations in CRC and found that APC-mutated tumours with malfunctioning PARK2 have a rapidly evolving carcinogenesis^39^.

The *ATR* gene, found mutated in a patient with colorectal cancer at very early ages, is considered as a hallmark of cancer^40^ and a component of the Fanconi Anaemia repair pathway^17^. Malfunctioning of the Fanconi anaemia pathway has been cited as a predisposing factor for CRC^13^. Interestingly, the *ATM* gene, which works together with *ATR* to maintain chromosome integrity and genome stability, has been considered as a moderately penetrant germline CRC predisposing gene^41^, suggesting that pathogenic alterations in *ATR* could also lead to a moderate CRC increased risk. Additionally, germline variants in *ATR* have already been associated with oropharyngeal cancer, as investigated by Tanaka et al. in a family with an *ATR*-related syndrome affecting 24 members from five generations^42^.

A missense alteration at *TREX1* also called our attention. The missense alteration identified was classified as pathogenic according to the ACMG criteria. Few studies related *TREX1* to cancer. Prati and collaborators detected an upregulation of *TREX1* in HPV-transformed cell lines in precancerous lesions, carcinomas, and adenocarcinomas^43^. The authors also found that *TREX1* silencing could affect tumour growth by upregulating p53, indicating a possible contribution to tumour development. Dong and collaborators detected *TREX1* alterations in pancreatic adenocarcinomas and concluded that *TREX1* might have a role in its carcinogenesis^44,45^. However, to the best of our knowledge, there is no studies associating *TREX1* to hereditary cancer. Further studies are needed in order to prove or discard this association.

It is important to highlight that variants located at splicing consensus regions in *ATM*, *GSDMA*, *PTPRE* and *RAD51B* genes were identified and, although classified as VUS in this manuscript considering the current evidences available, should be closely monitored as more evidences in the literature can lead to an upgrade on the classification from VUS to likely pathogenic or pathogenic.

This study has some limitations. The initial investigation in our cohort was based on the protein function, prioritizing the analysis of a virtual panel of cancer-associated genes. Although this strategy may have restricted the results obtained it allowed the identification of potentially pathogenic variants in six unrelated families among the 34 evaluated. Besides, alterations in the number of copies were not evaluated by our analysis pipeline.

Additionally, polygenic risk factors were not evaluated in this study, which might justify the cases with a family history suggestive of a cancer predisposition syndrome that did not present pathogenic/likely pathogenic variants. Candidate genes identified in this work require further cohort validations. Moreover, LOH analysis in tumor tissue, affected families-segregation analysis and functional studies should be addressed in order to investigate its role in oncogenesis. In spite of that, this is the biggest Brazilian study evaluating patients at high-risk for hereditary colorectal cancer fulfilling criteria for the FCCTX and allowed the identification of pathogenic/likely pathogenic variants in about twenty percent of the patients evaluated.

## Patients and methods

### Institutional review board statement and informed consent statement

The study was reviewed and approved by Barretos Cancer Hospital’s Research Ethics Committee (approval numbers: 53417916.5.0000.5437 and 56164716.9.0000.5437). All research was performed in accordance with the Brazilian CEP/CONEP-system regulation. All participants were de-identified and provided their written informed consent to participate in this study.

### Patient selection

Thirty-nine patients from 34 families identified at the Oncogenetics Department of Barretos Cancer Hospital^46^ were included in the study after signing the informed consent. All participants were negative for LS, which diagnostic is composed by immunohistochemistry (IHC) for MLH1, MSH2, MSH6, and PMS2 proteins; *BRAF* (V600E) sequencing and microsatellite instability (MSI) evaluation by fragment analysis^47^. Besides, gene panel sequencing by NGS is performed for all patients with High MSI or altered IHC. For the purpose of the present study, only patients with tumours presenting normal IHC results and microsatellite stability (MSS) were invited to participate.

From a clinical point of view, all families have at least two colorectal tumours in the family and 47% of them fulfilled the Amsterdam I criteria^3^. Clinical, sociodemographic, and histopathological information were extracted from the patients´ medical charts. Family history was obtained through the Oncogenetics chart, and all the pedigrees were drawn using Progeny software (https://pedigree.progenygenetics.com/).

### Exome sequencing

Genomic DNA was extracted from peripheral blood using the QIAmp DNA Blood Mini Kit with the automated QIAcube platform (QIAGEN—Germany). All procedures were performed following the manufacturer’s instructions. DNA concentration was determined using Qubit dsDNAHS Assay Kit (Thermo Fisher Scientific—United States).

Exome sequencing (ES) was performed with the SOPHiA Whole Exome Solution version 1 kit (203,058 targeted regions, 40,907,213 base pairs, and 19,682 genes) (Sophia Genetics SA, Saint Sulpice, Switzerland) on a NovaSeq platform (Illumina San Diego, Canada) by SOPHiA Genetics (Sophia Genetics SA, Saint Sulpice, Switzerland).

### Variant calling

Sequence reads were mapped to the human reference genome (GRCh37/hg19) using the Burrows-Wheeler Aligner (BWA, version 0.7.17)^48^. Alignment files were pre-processed and single nucleotide and indel germline variants were called by the Genome Analysis Toolkit (GATK version 4.0.4.0)^49^. Variant files were filtered to exclude variants covered by < 10 reads or with variant allele fraction < 25%.

### Genes analysed

**Figure 1:**
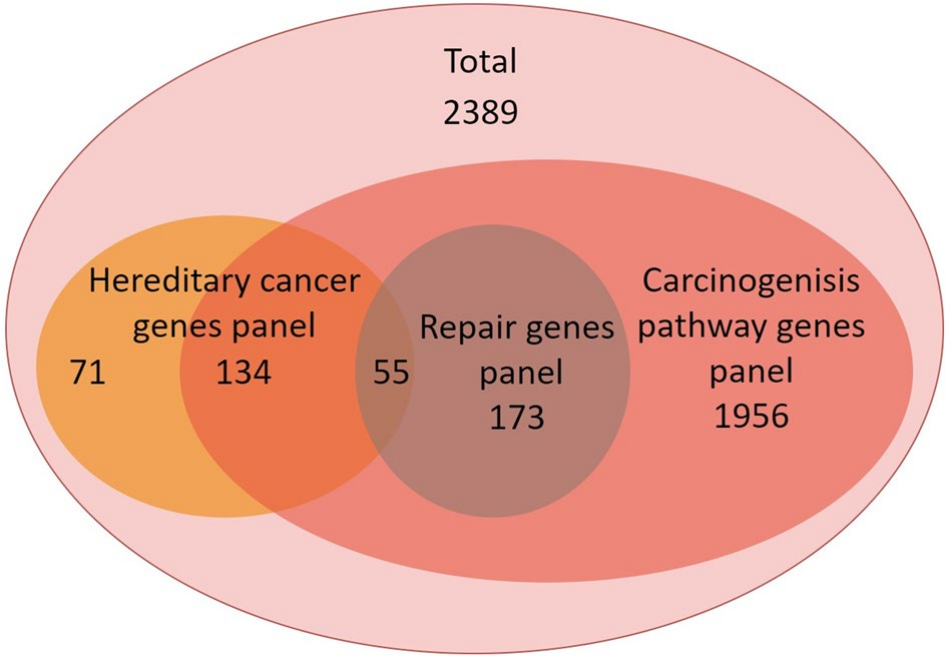
Venn diagram of the genes used in this study.

A virtual panel consisting of 2389 genes involved directly or indirectly in carcinogenesis was selected for analysis. This panel was extracted from the Catalogue of Somatic Mutations in Cancer^50^ (Cosmic: using the keyword terms “cancer”, “tumour-suppressor gene”, “proto-oncogene”, and “oncogene”), Universal Protein Resource (UniProt)^51^, and DISEASE study^52^. Additionally, a second “subpanel” consisting of 228 genes involved in DNA repair was extracted from the study by Das et al^17^. Finally, a third “subpanel” consisting of 260 genes related to hereditary cancer syndromes extracted from commercial panels and revised in the literature (GeneCard^19^ and Genetics Home Reference—Source: MedlinePlus, National Library of Medicine) was employed. The interactions of the gene panels are shown in Fig. 1 and the gene list evaluated is available in Table S1.

### Variant annotation and classification

**Figure 2:**
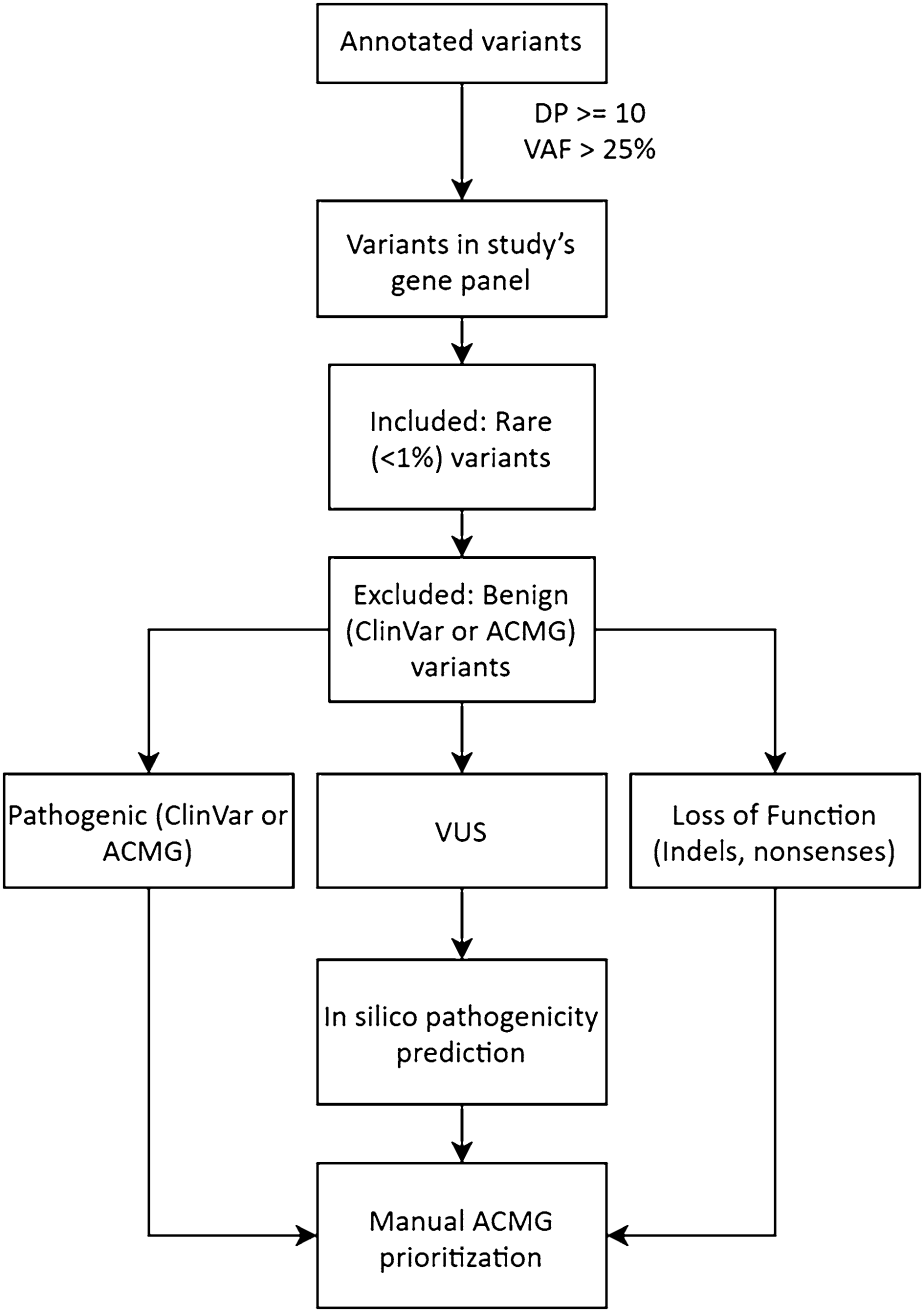
Variant prioritization decision pipeline.

To variant classification we developed a pipeline (Fig. 2) of decisions based on criteria proposed by the American College of Medical Genetics and Genomics (ACMG)^53^. Functional and populational frequency annotation of variants was performed using ANNOVAR^54^.

All variants covered by ≥ 10 reads, variant allele fraction ≥ 25%, and present in any gene of the three panels were first filtered by their populational frequency using two databases, ABraOM^55^ and gnomAD^56^: only variants with a minor allele frequency (MAF) < 1% were considered, with the exception of families with consanguinity, where we analysed variants with MAF ≥ 1%, although no class III, IV, or V variants were detected. Variants considered “variants of unknown significance” (VUS) according to the ACMG criteria were then filtered using the in silico pathogenicity prediction tool Rare Exome Variant Ensemble Learner (REVEL—score: 0.7)^57^ or Mendelian Clinically Applicable Pathogenicity (M-CAP—score: 0.025)^58^ for missense variants and Human Splicing Finder (HSF)^59^ for splicing variants. Additionally, Eigen (score: 0.7)^60^ and GenoCanyon (0.7)^61^ were used as complementary scores when the above tools were not available or as tiebreaker criteria.

### Variant manual review

Following the described automated prioritization steps, all variants classified as class III, IV, or V were manually curated in an independent way by two researchers (F.A.O.G and E.I.P). For this process, the ACMG criteria conferred by the Intervar^62^ and Varsome^63^, the GeneCard^19^ database, scientific literature (through PubMed—https://pubmed.ncbi.nlm.nih.gov/), and evidence deposited in the Clinvar^64^ database were reviewed. To avoid false positives, the variants were verified in the original BAM file using the Integrative Genome Viewer (IGV)^65^. For all analyses, we used the canonical transcript available at Locus Reference Genomic (LRG)^66^ or, if not available, the first one at Ensembl (http://www.ensembl.org/index.html).

### Conventional (Sanger) sequencing

Variants classified as likely pathogenic (ACMG class IV) or pathogenic (ACMG class V) were confirmed by bidirectional Sanger sequencing. For this process, we amplified the patients’ genomic DNA by PCR and purified the reaction with ExoSAP-IT (USB) enzyme and later with the BigDye Terminator kit (Thermo Fisher Scientific—United States). The sequencing itself was performed bidirectionally using the X-terminator kit v3.1 (Thermo Fisher Scientific—United States) on an automated sequencer model 3500 (Applied Biosystem—Thermo Fisher Scientific—United States).

## Conclusion

In conclusion, the present study represents the largest ES study involving Brazilian families at risk for FCCTX that allowed the identification of new candidate genes for FCCTX syndrome. In our cohort, 17.65% of our families were carriers of pathogenic/likely pathogenic variants that could explain cancer’s personal and family history. Our findings suggest that several cancer-associated genes may have a role in FCCTX, such as the known hereditary cancer gene *CHEK2*, the previously FCCTX candidate genes *OGG1* and *FAN1* and, other cancer-related genes such as *ATR, ASXL1, PARK2, SLX4* and *TREX1,* bringing novel insights into the genetic risk factors for familial colorectal cancer type X. Nevertheless, more studies (in vitro and/or in vivo*)*, such as functional assays, segregation, and loss of heterozygosity, are necessary to ascertain more conclusive hypotheses about their role in FCCTX predisposition.

## Supplementary Information

Supplementary Information.

## Data Availability

Class III, IV, and V variants identified in the 2389 evaluated genes shall be available in the ClinVar database; otherwise, the datasets presented in this article are not readily available due to privacy and ethical restrictions. Requests to access the datasets should be directed to the corresponding author.
