# Supplementary material for: New insights on familial colorectal cancer type X syndrome
Source: Sci Rep. 2022 Feb 18;12:2846. doi: 10.1038/s41598-022-06782-8 (PMC8857274; doi:10.1038/s41598-022-06782-8)

| **Table S1 - Cancer gene reference lists** | |
| --- | --- |
| **Gene Symbol** | **Hallmark** |
| 1433S | No |
| 2AAB | No |
| A1BG | No |
| A1CF | No |
| AAPK1 | No |
| ABCB1 | No |
| ABCG2 | No |
| ABI1 | Yes |
| ABI2 | No |
| ABL1 | Yes |
| ABL2 | No |
| ABLM3 | No |
| ABRX1 | No |
| ABTB1 | No |
| ACACA | No |
| ACBD4 | No |
| ACK1 | No |
| ACKR3 | Yes |
| ACL6A | No |
| ACPH | No |
| ACS2B | No |
| ACSL3 | Yes |
| ACSL5 | No |
| ACSL6 | No |
| ACTB | No |
| ACTN4 | No |
| ACTZ | No |
| ACV1B | No |
| ACVL1 | No |
| ACVR1 | Yes |
| ACVR2A | No |
| ADAM17 | No |
| ADAP1 | No |
| AEN | No |
| AF10 | No |
| AF17 | No |
| AF1Q | No |
| AF9 | No |
| AFAD | No |
| AFF1 | Yes |
| AFF3 | Yes |
| AFF4 | Yes |
| AFP | No |
| AGAP2 | No |
| AGR2 | No |
| AGRA2 | No |
| AGRA3 | No |
| AGRB1 | No |
| AGRF1 | No |
| AGRF2 | No |
| AGRL2 | No |
| AHRR | No |
| AIFM2 | No |
| AIM2 | No |
| AIP | No |
| AKAP9 | No |
| AKIP1 | No |
| AKP13 | No |
| AKT1 | Yes |
| AKT2 | No |
| AKT3 | No |
| ALB | No |
| ALDH2 | No |
| ALEX | No |
| ALK | Yes |
| ALKBH2 | No |
| ALKBH3 | No |
| ALPK1 | No |
| AMER1 | Yes |
| AMGO2 | No |
| AN32A | No |
| ANCHR | No |
| ANDR | No |
| ANK1 | No |
| ANM3 | No |
| ANM6 | No |
| ANO1 | No |
| ANS1A | No |
| ANS1B | No |
| ANXA5 | No |
| APC | Yes |
| APC10 | No |
| APCL | No |
| APEX1 | No |
| APEX2 | No |
| APITD1 | No |
| APLF | No |
| APOBEC3B | Yes |
| APTX | No |
| AR | Yes |
| ARAF | No |
| ARF | No |
| ARG39 | No |
| ARHG1 | No |
| ARHG4 | No |
| ARHG5 | No |
| ARHG8 | No |
| ARHGAP26 | No |
| ARHGAP5 | No |
| ARHGC | No |
| ARHGEF10 | No |
| ARHGEF10L | No |
| ARHGEF12 | No |
| ARHGI | No |
| ARHGQ | No |
| ARI3A | No |
| ARI3B | No |
| ARI4A | No |
| ARI5B | No |
| ARID1A | Yes |
| ARID1B | No |
| ARID2 | Yes |
| ARK73 | No |
| ARK74 | No |
| ARNT | Yes |
| ASC | No |
| ASF1A | No |
| ASPC1 | No |
| ASPP2 | No |
| ASPSCR1 | Yes |
| ASXL1 | Yes |
| ASXL2 | No |
| ATAD2 | No |
| ATF1 | Yes |
| ATF5 | No |
| ATF7 | No |
| ATIC | Yes |
| ATM | Yes |
| ATP1A1 | Yes |
| ATP2B3 | Yes |
| ATR | Yes |
| ATRIP | No |
| ATRX | Yes |
| ATXN3 | No |
| AURKA | No |
| AURKB | No |
| AXIN1 | Yes |
| AXIN2 | Yes |
| B2CL2 | No |
| B2L10 | No |
| B2LA1 | No |
| B2M | Yes |
| BACH2 | No |
| BAD | No |
| BAG3 | No |
| BAK | No |
| BANP | No |
| BAP1 | Yes |
| BARD1 | No |
| BATF | No |
| BATF2 | No |
| BATF3 | No |
| BAX | No |
| BAZ1A | No |
| BC11A | No |
| BC11B | No |
| BCAR1 | No |
| BCAS3 | No |
| BCAS4 | No |
| BCCIP | No |
| BCL10 | Yes |
| BCL11A | Yes |
| BCL11B | Yes |
| BCL2 | No |
| BCL2L1 | No |
| BCL2L12 | No |
| BCL3 | No |
| BCL6 | No |
| BCL6B | No |
| BCL7A | No |
| BCL9 | Yes |
| BCL9L | No |
| BCLAF1 | No |
| BCOR | Yes |
| BCORL1 | Yes |
| BCR | No |
| BECN1 | No |
| BIK | No |
| BIN1 | No |
| BIRC3 | Yes |
| BIRC6 | No |
| BLCAP | No |
| BLK | No |
| BLM | Yes |
| BLNK | No |
| BMI1 | No |
| BMP5 | No |
| BMPR1A | Yes |
| BMR1A | No |
| BMR1B | No |
| BMX | No |
| BOREA | No |
| BRAF | Yes |
| BRCA1 | Yes |
| BRCA2 | Yes |
| BRCC3 | No |
| BRD1 | No |
| BRD3 | No |
| BRD4 | Yes |
| BRD7 | No |
| BRD8 | No |
| BRI3B | No |
| BRIP1 | Yes |
| BRMS1 | No |
| BRNP1 | No |
| BTBD12 | No |
| BTC | No |
| BTG1 | No |
| BTG2 | No |
| BTG3 | No |
| BTK | Yes |
| BUB1B | Yes |
| C15orf65 | No |
| C17orf70 | No |
| C19orf40 | No |
| C1orf86 | No |
| C1QBP | No |
| C1TC | No |
| C2D1A | No |
| C2orf44 | No |
| C56D2 | No |
| C7orf11 | No |
| CA052 | No |
| CA053 | No |
| CA2D2 | No |
| CA2D3 | No |
| CACNA1D | Yes |
| CADH1 | No |
| CADH2 | No |
| CADH3 | No |
| CADM1 | No |
| CADM3 | No |
| CADM4 | No |
| CAH9 | No |
| CALCA | No |
| CALR | Yes |
| CAMTA1 | Yes |
| CANT1 | Yes |
| CARD11 | Yes |
| CARL3 | No |
| CARS | Yes |
| CASC3 | No |
| CASC5 | Yes |
| CASL | No |
| CASP2 | No |
| CASP3 | No |
| CASP6 | No |
| CASP8 | Yes |
| CASP9 | No |
| CASPA | No |
| CASR | No |
| CAV1 | No |
| CAVN3 | No |
| CBFA2T3 | No |
| CBFB | Yes |
| CBL | Yes |
| CBLB | Yes |
| CBLC | No |
| CBLL2 | No |
| CBP | No |
| CBX8 | No |
| CC85B | No |
| CCAR2 | No |
| CCD26 | No |
| CCD34 | No |
| CCDB1 | No |
| CCDC6 | Yes |
| CCL2 | No |
| CCNB1 | No |
| CCNB1IP1 | Yes |
| CCNC | No |
| CCND1 | Yes |
| CCND2 | Yes |
| CCND3 | No |
| CCNE1 | Yes |
| CCNE2 | No |
| CCNG1 | No |
| CCNG2 | No |
| CCNH | No |
| CCNL1 | No |
| CCNT2 | No |
| CCR4 | No |
| CCR7 | No |
| CD19 | No |
| CD209 | No |
| CD274 | No |
| CD28 | No |
| CD34 | No |
| CD38 | No |
| CD3Z | No |
| CD40LG | No |
| CD44 | No |
| CD5 | No |
| CD74 | No |
| CD79A | Yes |
| CD79B | Yes |
| CDC23 | No |
| CDC37 | No |
| CDC42 | No |
| CDC73 | Yes |
| CDCP1 | No |
| CDH1 | Yes |
| CDH10 | No |
| CDH11 | Yes |
| CDH17 | No |
| CDH2 | No |
| CDK1 | No |
| CDK10 | No |
| CDK12 | Yes |
| CDK2 | No |
| CDK20 | No |
| CDK4 | Yes |
| CDK6 | Yes |
| CDK7 | No |
| CDK9 | No |
| CDKA1 | No |
| CDKL1 | No |
| CDKL2 | No |
| CDKN1A | No |
| CDKN1B | No |
| CDKN1C | No |
| CDKN2A | Yes |
| CDKN2C | No |
| CDKN3 | No |
| CDN1A | No |
| CDN1B | No |
| CDN1C | No |
| CDN2A | No |
| CDN2B | No |
| CDN2C | No |
| CDN2D | No |
| CDON | No |
| CDT1 | No |
| CDX2 | No |
| CE162 | No |
| CEACAM5 | No |
| CEAM1 | No |
| CEAM5 | No |
| CEAM6 | No |
| CEBPA | No |
| CEBPG | No |
| CENPK | No |
| CENPU | No |
| CENPW | No |
| CEP57 | No |
| CEP89 | No |
| CETN2 | No |
| CHAF1A | No |
| CHAF1B | No |
| CHCHD7 | No |
| CHD1L | No |
| CHD2 | No |
| CHD4 | Yes |
| CHD5 | No |
| CHDH | No |
| CHEK1 | No |
| CHEK2 | Yes |
| CHFR | No |
| CHGA | No |
| CHIC2 | No |
| CHK1 | No |
| CHK2 | No |
| CHST11 | No |
| CHSTB | No |
| CIB1 | No |
| CIC | Yes |
| CIITA | Yes |
| CIP1 | No |
| CIP2A | No |
| CJ090 | No |
| CK095 | No |
| CKAP2 | No |
| CLD7 | No |
| CLIP1 | Yes |
| CLK2 | No |
| CLOCK | No |
| CLP1 | No |
| CLSPN | No |
| CLTC | Yes |
| CLTCL1 | Yes |
| CLUA1 | No |
| CLUS | No |
| CMC4 | No |
| CMTA1 | No |
| CNBD1 | No |
| CNBP | Yes |
| CNOT3 | Yes |
| CNTNAP2 | No |
| CNTRL | No |
| COL1A1 | No |
| COL2A1 | No |
| COL3A1 | No |
| COMD1 | No |
| COP1 | No |
| COX1 | No |
| COX6C | No |
| CPEB3 | No |
| CPEB4 | No |
| CPNE1 | No |
| CPNE3 | No |
| CQ080 | No |
| CR032 | No |
| CR3L2 | No |
| CR3L3 | No |
| CRADD | No |
| CRBL2 | No |
| CRCM | No |
| CREB1 | No |
| CREB3L1 | Yes |
| CREB3L2 | Yes |
| CREB5 | No |
| CREBBP | Yes |
| CREG1 | No |
| CRK | No |
| CRKL | No |
| CRLF2 | Yes |
| CRNKL1 | No |
| CRPAK | No |
| CRTC1 | Yes |
| CRTC3 | No |
| CRY1 | No |
| CSDE1 | No |
| CSF1R | No |
| CSF2 | No |
| CSF3 | No |
| CSF3R | No |
| CSK | No |
| CSK21 | No |
| CSK22 | No |
| CSK23 | No |
| CSMD3 | No |
| CSN3 | No |
| CSN5 | No |
| CSN6 | No |
| CSPP1 | No |
| CSRN1 | No |
| CTBP1 | No |
| CTCF | Yes |
| CTDS2 | No |
| CTDSL | No |
| CTIP | No |
| CTLA4 | No |
| CTNB1 | No |
| CTND1 | No |
| CTNNA1 | No |
| CTNNA2 | No |
| CTNNB1 | No |
| CTNND1 | No |
| CTNND2 | No |
| CUL1 | No |
| CUL2 | No |
| CUL3 | No |
| CUL4A | No |
| CUL4B | No |
| CUL5 | No |
| CUL7 | No |
| CUX1 | Yes |
| CUZD1 | No |
| CXCL12 | No |
| CXCR4 | Yes |
| CXXC5 | No |
| CYCS | No |
| CYLD | No |
| CYP19A1 | No |
| CYP1A1 | No |
| CYP2C8 | No |
| CYR61 | No |
| CYSLTR2 | No |
| CYTSB | No |
| DAB2 | No |
| DAB2P | No |
| DACH1 | No |
| DACT1 | No |
| DAPK2 | No |
| DAPK3 | No |
| DAXX | Yes |
| DCAF1 | No |
| DCAF12L2 | No |
| DCBD2 | No |
| DCC | No |
| DCLRE1A | No |
| DCLRE1B | No |
| DCLRE1C | No |
| DCNL1 | No |
| DCNL3 | No |
| DCR1B | No |
| DCTN1 | No |
| DDB1 | No |
| DDB2 | Yes |
| DDIT3 | Yes |
| DDIT4 | No |
| DDR1 | No |
| DDR2 | Yes |
| DDX10 | Yes |
| DDX17 | No |
| DDX3X | Yes |
| DDX41 | No |
| DDX42 | No |
| DDX5 | Yes |
| DDX6 | Yes |
| DEC1 | No |
| DEDD | No |
| DEDD2 | No |
| DEF1 | No |
| DEK | No |
| DEMA | No |
| DENR | No |
| DEP1A | No |
| DFFB | No |
| DGAT2 | No |
| DGCR14 | No |
| DGCR8 | No |
| DHB13 | No |
| DHSD | No |
| DI3L2 | No |
| DICER | No |
| DICER1 | Yes |
| DIRA3 | No |
| DIS3L2 | No |
| DJC27 | No |
| DKC1 | No |
| DKK1 | No |
| DKK2 | No |
| DKK3 | No |
| DKK4 | No |
| DLEC1 | No |
| DLG1 | No |
| DLG3 | No |
| DLGP5 | No |
| DLP1 | No |
| DMAP1 | No |
| DMBT1 | No |
| DMC1 | No |
| DMTF1 | No |
| DNAJB1 | No |
| DNM2 | Yes |
| DNMT3A | No |
| DNTT | No |
| DOCK4 | No |
| DOCK8 | No |
| DP13A | No |
| DPH1 | No |
| DPM3 | No |
| DPOD1 | No |
| DPOE1 | No |
| DPOLQ | No |
| DROSHA | Yes |
| DUS10 | No |
| DUS16 | No |
| DUS26 | No |
| DUS7 | No |
| DUT | No |
| DUX4L1 | No |
| E2F1 | No |
| E2F2 | No |
| E2F3 | No |
| E2F6 | No |
| E2F7 | No |
| E2F8 | No |
| E41L3 | No |
| E4F1 | No |
| EBF1 | Yes |
| ECT2 | No |
| ECT2L | No |
| EED | No |
| EFNA1 | No |
| EFNA3 | No |
| EFNA4 | No |
| EFNB3 | No |
| EFS | No |
| EGF | No |
| EGFR | Yes |
| EGLN1 | No |
| EGLN2 | No |
| EGR3 | No |
| EHBP1 | No |
| EHF | No |
| EI24 | No |
| EIF1AX | No |
| EIF3E | Yes |
| EIF4A2 | Yes |
| EKI2 | No |
| ELANE | No |
| ELF3 | No |
| ELF4 | Yes |
| ELF5 | No |
| ELK1 | No |
| ELK4 | Yes |
| ELL | Yes |
| ELN | No |
| ELOC | No |
| EME1 | No |
| EME2 | No |
| EML4 | Yes |
| EMP2 | No |
| EN113 | No |
| ENDOV | No |
| ENG | No |
| ENK18 | No |
| ENK19 | No |
| ENK21 | No |
| ENK24 | No |
| ENK5 | No |
| ENK6 | No |
| ENK7 | No |
| ENK8 | No |
| ENK9 | No |
| ENL | No |
| ENO2 | No |
| ENTP5 | No |
| EP300 | Yes |
| EPAS1 | Yes |
| EPC1 | No |
| EPCAM | No |
| EPHA2 | No |
| EPHA3 | No |
| EPHA4 | No |
| EPHA5 | No |
| EPHA7 | No |
| EPHA8 | No |
| EPHB2 | No |
| EPHB3 | No |
| EPHB6 | No |
| EPN1 | No |
| EPS15 | Yes |
| EPS8 | No |
| ERBB2 | Yes |
| ERBB3 | Yes |
| ERBB4 | Yes |
| ERC1 | Yes |
| ERCC1 | No |
| ERCC2 | Yes |
| ERCC3 | Yes |
| ERCC4 | Yes |
| ERCC5 | Yes |
| ERCC6 | No |
| ERCC6L | No |
| ERCC8 | No |
| ERD21 | No |
| EREG | No |
| ERF | No |
| ERG | Yes |
| ERRFI | No |
| ESCO2 | No |
| ESR1 | Yes |
| ESR2 | No |
| ESX1 | No |
| ETNK1 | Yes |
| ETS1 | No |
| ETS2 | No |
| ETV1 | No |
| ETV3 | No |
| ETV4 | No |
| ETV5 | No |
| ETV6 | Yes |
| ETV7 | No |
| EVI2A | No |
| EVI2B | No |
| EVI5 | No |
| EWS | No |
| EWSR1 | Yes |
| EXO1 | No |
| EXT1 | No |
| EXT2 | No |
| EYA2 | No |
| EZH2 | No |
| EZR | Yes |
| F107A | No |
| F10A5 | No |
| F120A | No |
| F189B | No |
| F210B | No |
| FA32A | No |
| FA58B | No |
| FA72A | No |
| FA83A | No |
| FA83B | No |
| FA83D | No |
| FABP7 | No |
| FACD2 | No |
| FADD | No |
| FAK1 | No |
| FAM131B | No |
| FAM135B | No |
| FAM175A | No |
| FAM3C | No |
| FAM46C | No |
| FAM47C | No |
| FAN1 | No |
| FANCA | No |
| FANCB | No |
| FANCC | No |
| FANCD2 | Yes |
| FANCE | No |
| FANCF | No |
| FANCG | No |
| FANCI | No |
| FANCJ | No |
| FANCL | No |
| FANCM | No |
| FAS | Yes |
| FASLG | No |
| FAT1 | Yes |
| FAT3 | No |
| FAT4 | Yes |
| FBLN1 | No |
| FBLN2 | No |
| FBSP1 | No |
| FBW1A | No |
| FBW1B | No |
| FBX5 | No |
| FBX7 | No |
| FBXL2 | No |
| FBXO11 | No |
| FBXW7 | Yes |
| FCG2B | No |
| FCGR2B | Yes |
| FCRL4 | No |
| FCSD2 | No |
| FEM1B | No |
| FEN1 | No |
| FER | No |
| FES | No |
| FEV | No |
| FGF1 | No |
| FGF10 | No |
| FGF2 | No |
| FGF22 | No |
| FGF3 | No |
| FGF4 | No |
| FGF5 | No |
| FGF6 | No |
| FGF7 | No |
| FGF8 | No |
| FGFP1 | No |
| FGFR1 | Yes |
| FGFR1OP | No |
| FGFR2 | Yes |
| FGFR3 | Yes |
| FGFR4 | Yes |
| FGOP2 | No |
| FGR | No |
| FH | No |
| FHIT | Yes |
| FHL2 | No |
| FIP1L1 | Yes |
| FKBP9 | No |
| FLCN | No |
| FLI1 | No |
| FLNA | No |
| FLOT1 | No |
| FLT1 | No |
| FLT3 | Yes |
| FLT3L | No |
| FLT4 | Yes |
| FNBP1 | No |
| FNIP1 | No |
| FNIP2 | No |
| FOLH1 | No |
| FOS | No |
| FOSL1 | No |
| FOSL2 | No |
| FOXA1 | Yes |
| FOXD1 | No |
| FOXE1 | No |
| FOXL2 | No |
| FOXO1 | No |
| FOXO3 | No |
| FOXO4 | No |
| FOXP1 | No |
| FOXR1 | No |
| FR1OP | No |
| FRAT1 | No |
| FRK | No |
| FRMD3 | No |
| FSCN1 | No |
| FSTL3 | No |
| FUBP1 | Yes |
| FUMH | No |
| FUND2 | No |
| FURIN | No |
| FUS | No |
| FYN | No |
| FZD1 | No |
| FZR1 | No |
| G3BP1 | No |
| GA45G | No |
| GADD45A | No |
| GALNT12 | No |
| GAS7 | Yes |
| GATA1 | No |
| GATA2 | No |
| GATA3 | No |
| GCNT3 | No |
| GCR | No |
| GDS1 | No |
| GEN1 | No |
| GFI1 | No |
| GFI1B | No |
| GGA1 | No |
| GIPC1 | No |
| GIT1 | No |
| GLI1 | No |
| GLI2 | No |
| GLT12 | No |
| GML | No |
| GMPS | No |
| GNA11 | Yes |
| GNAQ | Yes |
| GNAS | Yes |
| GNAS2 | No |
| GNL3L | No |
| GO45 | No |
| GOGA5 | No |
| GOLGA5 | No |
| GOPC | No |
| GOT1B | No |
| GP15L | No |
| GP5 | No |
| GPAT3 | No |
| GPC3 | Yes |
| GPC5 | No |
| GPHN | No |
| GPHRA | No |
| GPKOW | No |
| GPS2 | No |
| GRAP | No |
| GRB10 | No |
| GRB2 | No |
| GRB7 | No |
| GREM1 | No |
| GRHL2 | No |
| GRIN2A | No |
| GRM3 | No |
| GRP1 | No |
| GRWD1 | No |
| GSDMA | No |
| GSDME | No |
| GSTM1 | No |
| GSTP1 | No |
| GTF2H1 | No |
| GTF2H2 | No |
| GTF2H3 | No |
| GTF2H4 | No |
| GTF2H5 | No |
| GUAA | No |
| H2AFX | No |
| H3F3A | Yes |
| H3F3B | No |
| HABP2 | No |
| HASP | No |
| HBA1 | No |
| HCK | No |
| HDAC1 | No |
| HDAC2 | No |
| HDGR2 | No |
| HEAT6 | No |
| HELQ | No |
| HEMK1 | No |
| HERC1 | No |
| HERPUD1 | No |
| HEXB | No |
| HEXI1 | No |
| HEXI2 | No |
| HEY1 | Yes |
| HGF | No |
| HIC1 | No |
| HIC2 | No |
| HIF1A | Yes |
| HIF3A | No |
| HIP1 | Yes |
| HIPK2 | No |
| HIST1H3B | No |
| HIST1H4I | No |
| HLA-A | No |
| HLF | No |
| HLTF | No |
| HMGA1 | No |
| HMGA2 | No |
| HMGB1 | No |
| HMGB2 | No |
| HMGN2P46 | No |
| HNF1A | Yes |
| HNF1B | No |
| HNRNPA2B1 | Yes |
| HOOK3 | No |
| HOP | No |
| HOT | No |
| HOXA11 | Yes |
| HOXA13 | No |
| HOXA9 | No |
| HOXB13 | No |
| HOXC11 | No |
| HOXC13 | No |
| HOXD11 | No |
| HOXD13 | No |
| HPGDS | No |
| HRAS | Yes |
| HS90B | No |
| HSP90AA1 | No |
| HSP90AB1 | No |
| HTAI2 | No |
| HTATIP2 | No |
| HUS1 | No |
| HUTI | No |
| HUTU | No |
| HXA9 | No |
| HXB13 | No |
| HYAL1 | No |
| HYAL3 | No |
| I17RB | No |
| IASPP | No |
| ID1 | No |
| ID3 | No |
| IDH1 | Yes |
| IDH2 | Yes |
| IER2 | No |
| IF16 | No |
| IF2B3 | No |
| IF4E | No |
| IF5A2 | No |
| IFFO1 | No |
| IFIX | No |
| IFM1 | No |
| IFM3 | No |
| IFNA1 | No |
| IFNG | No |
| IFRD2 | No |
| IGF1 | No |
| IGF1R | No |
| IGF2 | No |
| IGF2BP2 | No |
| IGH | Yes |
| IGK | No |
| IGL | No |
| IKBB | No |
| IKBKB | No |
| IKZF1 | No |
| IL10 | No |
| IL15 | No |
| IL18 | No |
| IL1B | No |
| IL2 | No |
| IL21R | No |
| IL24 | No |
| IL3RB | No |
| IL4RA | No |
| IL6 | No |
| IL6ST | Yes |
| IL7R | No |
| IL8 | No |
| ILK | No |
| ILKAP | No |
| ING1 | No |
| ING2 | No |
| ING3 | No |
| ING4 | No |
| INSL3 | No |
| INSM1 | No |
| INSR | No |
| INT6 | No |
| IRF1 | No |
| IRF3 | No |
| IRF4 | No |
| IRF7 | No |
| IRS1 | No |
| IRS4 | No |
| ISK1 | No |
| IST1 | No |
| ISX | No |
| ITA9 | No |
| ITCH | No |
| ITGAV | No |
| ITK | No |
| ITPA | No |
| IWS1 | No |
| JAK1 | Yes |
| JAK2 | Yes |
| JAK3 | Yes |
| JAZF1 | No |
| JIP4 | No |
| JTB | No |
| JUN | No |
| JUND | No |
| JUPI2 | No |
| KANK1 | No |
| KAP0 | No |
| KAPCB | No |
| KAT2A | No |
| KAT2B | No |
| KAT5 | No |
| KAT6A | No |
| KAT6B | No |
| KAT7 | No |
| KC1D | No |
| KC1E | No |
| KC1G2 | No |
| KCC4 | No |
| KCD11 | No |
| KCD21 | No |
| KCNA1 | No |
| KCNJ5 | No |
| KCTD6 | No |
| KDM1A | No |
| KDM3B | No |
| KDM5A | No |
| KDM5C | No |
| KDM6A | Yes |
| KDR | Yes |
| KDSR | No |
| KEAP1 | Yes |
| KHDR1 | No |
| KI20B | No |
| KIAA1549 | No |
| KIAA1598 | No |
| KIF1B | No |
| KIF22 | No |
| KIF5B | No |
| KILIN | No |
| KIME | No |
| KISS1 | No |
| KIT | Yes |
| KLF4 | Yes |
| KLF5 | No |
| KLF6 | No |
| KLH20 | No |
| KLH22 | No |
| KLH41 | No |
| KLK10 | No |
| KLK2 | No |
| KLK3 | No |
| KMT2A | No |
| KMT2B | No |
| KMT2C | No |
| KMT2D | No |
| KMT2E | No |
| KMT5A | No |
| KNL1 | No |
| KNSTRN | No |
| KPCA | No |
| KPCD | No |
| KPCD2 | No |
| KPCI | No |
| KPCL | No |
| KPYM | No |
| KRAS | Yes |
| KRIT1 | No |
| KRT19 | No |
| KRT7 | No |
| KS6A2 | No |
| KS6A5 | No |
| KS6B1 | No |
| KS6B2 | No |
| KSYK | No |
| KTN1 | No |
| L2GL1 | No |
| LACTB | No |
| LAP4B | No |
| LARP4B | No |
| LASP1 | No |
| LATS1 | No |
| LATS2 | No |
| LC7L3 | No |
| LCK | Yes |
| LCP1 | No |
| LDB1 | No |
| LEF1 | Yes |
| LEG1 | No |
| LEG8 | No |
| LEPROTL1 | No |
| LEU1 | No |
| LFG1 | No |
| LGI1 | No |
| LGR6 | No |
| LHFP | No |
| LHX2 | No |
| LHX4 | No |
| LIFR | Yes |
| LIG1 | No |
| LIG3 | No |
| LIG4 | No |
| LIMA1 | No |
| LIMD1 | No |
| LIMK1 | No |
| LIN7A | No |
| LIN9 | No |
| LITAF | No |
| LMBL1 | No |
| LMF1 | No |
| LMNA | Yes |
| LMO1 | No |
| LMO2 | No |
| LOX12 | No |
| LPP | No |
| LRIG3 | No |
| LRP12 | No |
| LRP1B | Yes |
| LRRN2 | No |
| LSM14A | No |
| LSM7 | No |
| LTK | No |
| LTMD1 | No |
| LUR1L | No |
| LUZP4 | No |
| LYL1 | No |
| LYN | No |
| LYOX | No |
| LZTR1 | Yes |
| LZTS1 | No |
| LZTS2 | No |
| M3K1 | No |
| M3K10 | No |
| M3K11 | No |
| M3K14 | No |
| M3K2 | No |
| M3K5 | No |
| M3K7 | No |
| M3K8 | No |
| M4K5 | No |
| MAD2L2 | No |
| MADD | No |
| MAEA | No |
| MAF | No |
| MAFA | No |
| MAFB | No |
| MAFF | No |
| MAFG | No |
| MAFK | No |
| MAGI1 | No |
| MAGI3 | No |
| MAK | No |
| MALAT1 | No |
| MALT1 | No |
| MAML2 | No |
| MANF | No |
| MAP12 | No |
| MAP2K1 | No |
| MAP2K2 | No |
| MAP2K4 | No |
| MAP2K7 | No |
| MAP3K1 | No |
| MAP3K13 | No |
| MAPK1 | Yes |
| MAPK3 | No |
| MAPK5 | No |
| MAPK8 | No |
| MAPK8IP1 | No |
| MAPK8IP2 | No |
| MARE1 | No |
| MARE3 | No |
| MAS | No |
| MATK | No |
| MATR3 | No |
| MAVS | No |
| MAX | No |
| MB21D2 | No |
| MBD4 | No |
| MBP | No |
| MC1R | No |
| MCF2 | No |
| MCF2L | No |
| MCL1 | No |
| MCM4 | No |
| MCTS1 | No |
| MD1L1 | No |
| MDC1 | No |
| MDGA1 | No |
| MDM2 | No |
| MDM4 | No |
| MDS2 | No |
| MECOM | No |
| MED1 | No |
| MED10 | No |
| MED12 | Yes |
| MED28 | No |
| MELK | No |
| MEN1 | No |
| MEP50 | No |
| MERL | No |
| MERTK | No |
| MET | No |
| MFHA1 | No |
| MFR1L | No |
| MGMT | No |
| MGP | No |
| MIB2 | No |
| MIEN1 | No |
| MINK1 | No |
| MINP1 | No |
| MINY3 | No |
| MITF | No |
| MK01 | No |
| MK03 | No |
| MK06 | No |
| MK07 | No |
| MK09 | No |
| MK15 | No |
| MKL1 | No |
| MKL2 | No |
| MKRN2 | No |
| MLF1 | No |
| MLH1 | No |
| MLH3 | No |
| MLLT1 | No |
| MLLT10 | No |
| MLLT11 | No |
| MLLT3 | No |
| MLLT4 | No |
| MLLT6 | No |
| MLX | No |
| MME | No |
| MMP1 | No |
| MMP14 | No |
| MMP2 | No |
| MMP9 | No |
| MMRN2 | No |
| MMS19 | No |
| MN1 | No |
| MNAT1 | No |
| MNX1 | No |
| MO4L1 | No |
| MO4L2 | No |
| MOB1A | No |
| MOD5 | No |
| MOS | No |
| MOV10 | No |
| MP2K1 | No |
| MP2K4 | No |
| MP2K6 | No |
| MP2K7 | No |
| MPG | No |
| MPIP2 | No |
| MPIP3 | No |
| MPL | No |
| MPP3 | No |
| MRE11 | No |
| MRE11A | No |
| MRGBP | No |
| MRVI1 | No |
| MSD3 | No |
| MSH2 | No |
| MSH3 | No |
| MSH4 | No |
| MSH5 | No |
| MSH6 | No |
| MSI2 | No |
| MSMB | No |
| MSN | No |
| MSRE | No |
| MTA1 | No |
| MTA2 | No |
| MTA3 | No |
| MTCP1 | No |
| MTG16 | No |
| MTG8 | No |
| MTG8R | No |
| MTND | No |
| MTOR | Yes |
| MTSS1 | No |
| MTUS1 | No |
| MUC1 | No |
| MUC16 | No |
| MUC4 | No |
| MUL1 | No |
| MUS81 | No |
| MUTYH | No |
| MXI1 | No |
| MXRA5 | No |
| MY18B | No |
| MYB | No |
| MYBA | No |
| MYBB | No |
| MYC | Yes |
| MYCL | No |
| MYCN | No |
| MYCP1 | No |
| MYD88 | No |
| MYEOV | No |
| MYH11 | No |
| MYH9 | No |
| MYO5A | No |
| MYOD1 | No |
| N4BP2 | No |
| NAA15 | No |
| NAB2 | Yes |
| NACA | No |
| NACC2 | No |
| NADAP | No |
| NANO1 | No |
| NANP8 | No |
| NARR | No |
| NAT6 | No |
| NAV2 | No |
| NBAS | No |
| NBEA | No |
| NBL1 | No |
| NBN | No |
| NBPF3 | No |
| NBPFC | No |
| NCAM1 | No |
| NCKIPSD | No |
| NCOA1 | No |
| NCOA2 | No |
| NCOA4 | No |
| NCOR1 | No |
| NCOR2 | Yes |
| NDC80 | No |
| NDE1 | No |
| NDEL1 | No |
| NDKA | No |
| NDKB | No |
| NDRG1 | Yes |
| NDRG2 | No |
| NDUAD | No |
| NDUC2 | No |
| NDUF4 | No |
| NEB2 | No |
| NEDD4 | No |
| NEDD8 | No |
| NEIL1 | No |
| NEIL2 | No |
| NEIL3 | No |
| NEK2 | No |
| NEK3 | No |
| NEK4 | No |
| NEMF | No |
| NEMO | No |
| NENF | No |
| NEO1 | No |
| NEUL1 | No |
| NF1 | Yes |
| NF2 | Yes |
| NFAT5 | No |
| NFATC2 | No |
| NFE2L2 | Yes |
| NFIB | No |
| NFIP1 | No |
| NFIP2 | No |
| NFKB1 | No |
| NFKB2 | No |
| NFKBIE | No |
| NGAL | No |
| NGF | No |
| NHEJ1 | No |
| NIN | No |
| NINL | No |
| NKX21 | No |
| NKX2-1 | No |
| NKX31 | No |
| NKX3-1 | No |
| NLS1 | No |
| NMI | No |
| NOL11 | No |
| NOL7 | No |
| NONO | Yes |
| NOP53 | No |
| NOTC4 | No |
| NOTCH1 | No |
| NOTCH2 | No |
| NOV | No |
| NPAT | No |
| NPM | No |
| NPM1 | No |
| NPRL2 | No |
| NR4A3 | No |
| NRAS | Yes |
| NRG1 | No |
| NS1BP | No |
| NSA2 | No |
| NSD1 | No |
| NSD2 | No |
| NSD3 | No |
| NT5C2 | No |
| NTHL1 | No |
| NTRK1 | No |
| NTRK3 | No |
| NU214 | No |
| NUAK1 | No |
| NUDT1 | No |
| NUMA1 | No |
| NUP214 | No |
| NUP98 | No |
| NUTM1 | No |
| NUTM2A | No |
| NUTM2B | No |
| OBF1 | No |
| OBFC2B | No |
| OBSCN | No |
| OGG1 | No |
| OGR1 | No |
| OLIG2 | No |
| OMD | No |
| OPCM | No |
| OTU7B | No |
| P2RY8 | No |
| P3H1 | No |
| P53 | No |
| P73 | No |
| P85A | No |
| P85B | No |
| PA216 | No |
| PA2G4 | No |
| PABPC1 | No |
| PAF1 | No |
| PAF15 | No |
| PAFAH1B2 | Yes |
| PAK1 | No |
| PAK4 | No |
| PAK5 | No |
| PAL4A | No |
| PALB2 | No |
| PALLD | No |
| PANO1 | No |
| PAR10 | No |
| PAR6A | No |
| PARK7 | No |
| PARN | No |
| PARP1 | No |
| PARP2 | No |
| PARP3 | No |
| PARP4 | No |
| PATZ1 | No |
| PAWR | No |
| PAX3 | Yes |
| PAX5 | Yes |
| PAX7 | Yes |
| PAX8 | Yes |
| PAXI | No |
| PB1 | No |
| PBIP1 | No |
| PBRM1 | No |
| PBX1 | Yes |
| PBX2 | No |
| PBX3 | No |
| PC11Y | No |
| PCA3 | No |
| PCBP1 | No |
| PCD15 | No |
| PCLI1 | No |
| PCM1 | No |
| PCNA | No |
| PCSK7 | No |
| PCX2 | No |
| PDCD1LG2 | Yes |
| PDCD4 | No |
| PDE4DIP | No |
| PDGFB | No |
| PDGFC | No |
| PDGFD | No |
| PDGFRA | No |
| PDGFRB | No |
| PDLI5 | No |
| PDPN | No |
| PDRG1 | No |
| PDZD4 | No |
| PEA15 | No |
| PEBB | No |
| PEG10 | No |
| PER1 | Yes |
| PERP | No |
| PFD3 | No |
| PGDH | No |
| PGFRA | No |
| PGFRB | No |
| PGFRL | No |
| PGM1 | No |
| PGR | No |
| PGRP2 | No |
| PHB | No |
| PHF23 | No |
| PHF6 | No |
| PHLA3 | No |
| PHLP1 | No |
| PHLP2 | No |
| PHOX2B | Yes |
| PIAS1 | No |
| PIAS2 | No |
| PIAS4 | No |
| PICAL | No |
| PICALM | Yes |
| PIDD1 | No |
| PIGU | No |
| PIK3CA | Yes |
| PIK3CB | Yes |
| PIK3R1 | Yes |
| PIM1 | Yes |
| PIM2 | No |
| PIM3 | No |
| PIMRE | No |
| PIN1 | No |
| PININ | No |
| PINK1 | No |
| PINX1 | No |
| PIR | No |
| PIWL1 | No |
| PIWL2 | No |
| PK3CA | No |
| PK3CB | No |
| PK3CD | No |
| PKHG2 | No |
| PKHG5 | No |
| PKHO1 | No |
| PLAG1 | No |
| PLAK | No |
| PLAL1 | No |
| PLCE1 | No |
| PLCG1 | No |
| PLGF | No |
| PLK1 | No |
| PLK2 | No |
| PLK3 | No |
| PLPL3 | No |
| PLPP5 | No |
| PLXB1 | No |
| PLXB2 | No |
| PLXB3 | No |
| PML | No |
| PMS1 | No |
| PMS2 | Yes |
| PMS2L3 | No |
| PNKP | No |
| PO3F2 | No |
| PO4F1 | No |
| PO4F2 | No |
| POLA1 | No |
| POLB | No |
| POLD1 | No |
| POLD2 | No |
| POLD3 | No |
| POLD4 | No |
| POLE | No |
| POLE2 | No |
| POLE3 | No |
| POLE4 | No |
| POLG | No |
| POLH | No |
| POLI | No |
| POLK | No |
| POLL | No |
| POLM | No |
| POLN | No |
| POLQ | No |
| POT1 | No |
| POU2AF1 | Yes |
| POU5F1 | Yes |
| PP1A | No |
| PPARG | Yes |
| PPFIBP1 | No |
| PPIE | No |
| PPM1D | Yes |
| PPP2R1A | No |
| PPP6C | Yes |
| PPR18 | No |
| PRAF3 | No |
| PRAM | No |
| PRC1 | No |
| PRCC | No |
| PRDM1 | No |
| PRDM16 | Yes |
| PRDM2 | No |
| PRDM5 | No |
| PRDX6 | No |
| PREX2 | Yes |
| PRF1 | No |
| PRKACA | Yes |
| PRKAR1A | Yes |
| PRKCB | No |
| PRKDC | No |
| PRKN | No |
| PRKX | No |
| PROM1 | No |
| PRPF19 | No |
| PRPF40B | No |
| PRR14 | No |
| PRR5 | No |
| PRRX1 | No |
| PRUN1 | No |
| PRUN2 | No |
| PSA5 | No |
| PSCA | No |
| PSD10 | No |
| PSIP1 | Yes |
| PSMD6 | No |
| PTC1 | No |
| PTC2 | No |
| PTCH1 | No |
| PTEN | Yes |
| PTGS2 | No |
| PTHB1 | No |
| PTK6 | Yes |
| PTK7 | No |
| PTN13 | No |
| PTN14 | No |
| PTN18 | No |
| PTN7 | No |
| PTOV1 | No |
| PTPN11 | No |
| PTPN11 | No |
| PTPN13 | Yes |
| PTPN6 | No |
| PTPRB | No |
| PTPRC | No |
| PTPRD | No |
| PTPRE | No |
| PTPRH | No |
| PTPRJ | No |
| PTPRK | No |
| PTPRN | No |
| PTPRO | No |
| PTPRT | Yes |
| PTPRU | No |
| PTTG1 | No |
| PTTG2 | No |
| PTTG3 | No |
| PUM1 | No |
| PUM2 | No |
| PWWP2A | No |
| PXMP4 | No |
| QKI | Yes |
| R144B | No |
| RA51C | No |
| RA51D | No |
| RA54B | No |
| RAB11B | No |
| RAB26 | No |
| RAB5A | No |
| RAB7A | No |
| RAB8A | No |
| RABEP1 | Yes |
| RAC1 | Yes |
| RACK1 | No |
| RAD1 | No |
| RAD17 | No |
| RAD18 | No |
| RAD21 | Yes |
| RAD23A | No |
| RAD23B | No |
| RAD50 | No |
| RAD51 | No |
| RAD51B | No |
| RAD51C | No |
| RAD51D | No |
| RAD52 | No |
| RAD54 | No |
| RAD54B | No |
| RAD54L | No |
| RAD9A | No |
| RAF1 | Yes |
| RAI3 | No |
| RALGDS | No |
| RANB9 | No |
| RANBP2 | Yes |
| RAP1A | No |
| RAP1GDS1 | Yes |
| RAP2A | No |
| RAP2B | No |
| RARA | No |
| RARB | No |
| RASA1 | No |
| RASF1 | No |
| RASF2 | No |
| RASF3 | No |
| RASF4 | No |
| RASF5 | No |
| RASF6 | No |
| RASFA | No |
| RASH | No |
| RASK | No |
| RASM | No |
| RASN | No |
| RB | No |
| RB1 | Yes |
| RB11A | No |
| RB6I2 | No |
| RBBP8 | No |
| RBCC1 | No |
| RBG1L | No |
| RBL1 | No |
| RBL2 | No |
| RBM10 | Yes |
| RBM14 | No |
| RBM15 | Yes |
| RBM5 | No |
| RBM6 | No |
| RBMS1 | No |
| RBMX | No |
| RBP56 | No |
| RBTN1 | No |
| RBTN2 | No |
| RBX1 | No |
| RBX2 | No |
| RDM1 | No |
| REC6 | No |
| RECK | No |
| RECQ4 | No |
| RECQL | No |
| RECQL4 | Yes |
| RECQL5 | No |
| RED | No |
| REL | No |
| REPS2 | No |
| RET | No |
| RETN | No |
| REV1L | No |
| REV3L | No |
| RFC1 | No |
| RFC2 | No |
| RFC3 | No |
| RFC4 | No |
| RFC5 | No |
| RFIP3 | No |
| RFWD3 | No |
| RGCC | No |
| RGDSR | No |
| RGPD3 | No |
| RGRF1 | No |
| RGS7 | No |
| RHBDF2 | No |
| RHDF2 | No |
| RHG07 | No |
| RHG20 | No |
| RHG21 | No |
| RHG26 | No |
| RHG29 | No |
| RHG35 | No |
| RHNO1 | No |
| RHOA | Yes |
| RHOB | No |
| RHOG | No |
| RHOH | Yes |
| RHXF2 | No |
| RIF1 | No |
| RIG | No |
| RINT1 | No |
| RIOX1 | No |
| RIOX2 | No |
| RIPK1 | No |
| RIT2 | No |
| RL10 | No |
| RL1D1 | No |
| RL40 | No |
| RL7A | No |
| RLF | No |
| RMI1 | No |
| RMI2 | No |
| RMP | No |
| RN135 | No |
| RN139 | No |
| RN149 | No |
| RN167 | No |
| RN213 | No |
| RN5A | No |
| RNF11 | No |
| RNF168 | No |
| RNF213 | Yes |
| RNF34 | No |
| RNF4 | No |
| RNF43 | Yes |
| RNF6 | No |
| RNF8 | No |
| RNT2 | No |
| RNZ2 | No |
| ROBO1 | No |
| ROBO2 | No |
| RON | No |
| ROR1 | No |
| RORA | No |
| ROS1 | Yes |
| RPA1 | No |
| RPA2 | No |
| RPA3 | No |
| RPA4 | No |
| RPB1 | No |
| RPL10 | No |
| RPL22 | No |
| RPL5 | No |
| RPN1 | No |
| RRAS2 | No |
| RRM2B | No |
| RRP1B | No |
| RS27A | No |
| RS30 | No |
| RSLAA | No |
| RSPO2 | No |
| RSPO3 | No |
| RSSA | No |
| RT11 | No |
| RTEL1 | No |
| RTKN | No |
| RTN4 | No |
| RUBCL | No |
| RUFY3 | No |
| RUNDC2A | No |
| RUNX1 | No |
| RUNX1T1 | No |
| RUNX2 | No |
| RUNX3 | No |
| RUVB1 | No |
| RUVB2 | No |
| RUXG | No |
| RXFP2 | No |
| RYK | No |
| S100A7 | No |
| S10A2 | No |
| S22AA | No |
| S22AI | No |
| S2533 | No |
| S2547 | No |
| S35B2 | No |
| S38A3 | No |
| SALL4 | No |
| SAMN1 | No |
| SAPC2 | No |
| SASH1 | No |
| SAV1 | No |
| SBDS | No |
| SC23B | No |
| SC31A | No |
| SC5A8 | No |
| SCRIB | No |
| SCUB3 | No |
| SDC1 | No |
| SDC4 | No |
| SDCB1 | No |
| SDCG3 | No |
| SDHA | Yes |
| SDHAF2 | No |
| SDHB | No |
| SDHC | No |
| SDHD | No |
| SE6L1 | No |
| SEM3B | No |
| SEM3F | No |
| SEM5A | No |
| SENP1 | No |
| SENP6 | No |
| SEPT5 | No |
| SEPT6 | No |
| SEPT9 | No |
| SESN1 | No |
| SESN2 | No |
| SET | No |
| SETBP | No |
| SETBP1 | No |
| SETD1B | No |
| SETD2 | No |
| SETMAR | No |
| SETMR | No |
| SF3B1 | No |
| SFPQ | Yes |
| SFRP4 | No |
| SG2A2 | No |
| SGK1 | No |
| SH21B | No |
| SH2B1 | No |
| SH2B2 | No |
| SH2B3 | No |
| SH3G1 | No |
| SH3GL1 | No |
| SH3R2 | No |
| SHB | No |
| SHC1 | No |
| SHC2 | No |
| SHC3 | No |
| SHCAF | No |
| SHFM1 | No |
| SHIP2 | No |
| SHPRH | No |
| SHSA5 | No |
| SIAH1 | No |
| SIK1 | No |
| SIK3 | No |
| SIR1 | No |
| SIR2 | No |
| SIR4 | No |
| SIRPA | No |
| SIVA | No |
| SIX1 | No |
| SIX2 | No |
| SKI | No |
| SKIL | No |
| SLAP2 | No |
| SLC34A2 | Yes |
| SLC45A3 | Yes |
| SLIP | No |
| SLX1A | No |
| SLX1B | No |
| SLX4 | No |
| SMAD2 | Yes |
| SMAD3 | Yes |
| SMAD4 | No |
| SMAD5 | No |
| SMAD6 | No |
| SMAD7 | No |
| SMAGP | No |
| SMARCA4 | No |
| SMARCB1 | No |
| SMARCD1 | No |
| SMARCE1 | No |
| SMC1A | No |
| SMC3 | No |
| SMC5 | No |
| SMCA4 | No |
| SMO | No |
| SMUF2 | No |
| SMUG1 | No |
| SNAI1 | No |
| SNAI2 | No |
| SND1 | No |
| SNF5 | No |
| SNIP1 | No |
| SNTB1 | No |
| SNW1 | No |
| SOCS1 | No |
| SOX2 | No |
| SOX21 | No |
| SP1 | No |
| SP100 | No |
| SPAG1 | No |
| SPECC1 | No |
| SPEN | No |
| SPI1 | No |
| SPN90 | No |
| SPO11 | No |
| SPOP | Yes |
| SPRTN | No |
| SPT13 | No |
| SQSTM | No |
| SRC | No |
| SRC8 | No |
| SRGAP3 | No |
| SRGP1 | No |
| SRSF1 | No |
| SRSF2 | No |
| SRSF3 | No |
| SRY | No |
| SS18 | No |
| SS18L1 | No |
| SSPN | No |
| SSX1 | No |
| SSX2 | No |
| SSX4 | No |
| SSXT | No |
| ST134 | No |
| ST14 | No |
| ST17A | No |
| ST18 | No |
| ST20 | No |
| ST5 | No |
| ST7 | No |
| ST7L | No |
| STA13 | No |
| STABP | No |
| STAG1 | No |
| STAG2 | No |
| STAP2 | No |
| STAR8 | No |
| STAT3 | No |
| STAT5B | No |
| STAT6 | No |
| STEA3 | No |
| STEA4 | No |
| STIL | No |
| STK11 | No |
| STK25 | No |
| STK26 | No |
| STK3 | No |
| STK38 | No |
| STK39 | No |
| STK4 | No |
| STRN | No |
| STYK1 | No |
| SUFU | No |
| SUMO1 | No |
| SUSD2 | No |
| SUSD3 | No |
| SUSD6 | No |
| SUV91 | No |
| SUV92 | No |
| SUZ12 | Yes |
| SYCC | No |
| SYCP3 | No |
| SYK | No |
| SYNP2 | No |
| SYP | No |
| SZRD1 | No |
| T184B | No |
| T53I1 | No |
| T53I2 | No |
| TACC1 | No |
| TACC2 | No |
| TACC3 | No |
| TAD2B | No |
| TAF12 | No |
| TAF15 | No |
| TAF4 | No |
| TAL1 | No |
| TAL2 | No |
| TAOK1 | No |
| TAOK2 | No |
| TAOK3 | No |
| TARG1 | No |
| TAXB1 | No |
| TBC3A | No |
| TBK1 | No |
| TBL1XR1 | Yes |
| TBP | No |
| TBRG1 | No |
| TBX3 | No |
| TCAL7 | No |
| TCAM1 | No |
| TCAM2 | No |
| TCEA1 | No |
| TCF12 | No |
| TCF3 | No |
| TCF7L2 | Yes |
| TCHP | No |
| TCL1A | Yes |
| TCL1B | No |
| TCP1L | No |
| TCTA | No |
| TDG | No |
| TDP1 | No |
| TDP2 | No |
| TEC | No |
| TEF | No |
| TEFF1 | No |
| TENS4 | No |
| TERC | No |
| TERT | Yes |
| TES | No |
| TET1 | Yes |
| TET2 | No |
| TEX10 | No |
| TF2H1 | No |
| TF65 | No |
| TF7L2 | No |
| TFDP1 | No |
| TFDP2 | No |
| TFDP3 | No |
| TFE2 | No |
| TFE3 | Yes |
| TFEB | No |
| TFG | No |
| TFIP8 | No |
| TFPT | No |
| TFRC | No |
| TG | No |
| TGFA | No |
| TGFB1 | No |
| TGFBR2 | Yes |
| TGFR1 | No |
| TGFR2 | No |
| THA | No |
| THADA | No |
| THAP1 | No |
| THB | No |
| THEM4 | No |
| THOC1 | No |
| THRAP3 | No |
| TIAM1 | No |
| TIE2 | No |
| TIF1A | No |
| TIFA | No |
| TINF2 | No |
| TIPRL | No |
| TISB | No |
| TISD | No |
| TLK1 | No |
| TLK2 | No |
| TLX1 | No |
| TLX3 | No |
| TM101 | No |
| TM102 | No |
| TM115 | No |
| TM127 | No |
| TM158 | No |
| TM9S4 | No |
| TMED4 | No |
| TMED8 | No |
| TMEM127 | No |
| TMF1 | No |
| TMPRSS2 | Yes |
| TNAP3 | No |
| TNC | No |
| TNF | No |
| TNF15 | No |
| TNFAIP3 | Yes |
| TNFRSF10B | No |
| TNFRSF14 | No |
| TNFRSF17 | No |
| TNFRSF8 | No |
| TNIP2 | No |
| TNIP3 | No |
| TNK1 | No |
| TNR17 | No |
| TNR1A | No |
| TNR6A | No |
| TNR6B | No |
| TNR6C | No |
| TOB1 | No |
| TOB2 | No |
| TOP1 | No |
| TOP3A | No |
| TOPB1 | No |
| TOPBP1 | No |
| TOPRS | No |
| TP4A2 | No |
| TP53 | Yes |
| TP53BP1 | No |
| TP63 | Yes |
| TPD52 | No |
| TPD53 | No |
| TPGS2 | No |
| TPM3 | No |
| TPM4 | No |
| TPO | No |
| TPOR | No |
| TPR | No |
| TR10B | No |
| TRA | No |
| TRADD | No |
| TRAF1 | No |
| TRAF3 | No |
| TRAF5 | No |
| TRAF6 | No |
| TRAF7 | No |
| TRB | No |
| TRD | No |
| TREX1 | No |
| TREX2 | No |
| TRG-GCC2 | No |
| TRI13 | No |
| TRI22 | No |
| TRI27 | No |
| TRI33 | No |
| TRI35 | No |
| TRI36 | No |
| TRI37 | No |
| TRIB3 | No |
| TRIM24 | No |
| TRIM27 | No |
| TRIM33 | No |
| TRIM8 | No |
| TRIP11 | No |
| TRRAP | No |
| TS101 | No |
| TSC1 | No |
| TSC2 | No |
| TSHR | No |
| TSN31 | No |
| TSN6 | No |
| TTC23 | No |
| TTK | No |
| TTP | No |
| TUSC1 | No |
| TUSC2 | No |
| TX1B3 | No |
| TXK | No |
| TXNIP | No |
| TYDP2 | No |
| TYK2 | No |
| TYRO | No |
| TYRO3 | No |
| TYW4 | No |
| TYY1 | No |
| TZAP | No |
| U2AF1 | No |
| U2QL1 | No |
| UB2D2 | No |
| UB2R1 | No |
| UB2R2 | No |
| UB2V2 | No |
| UBB | No |
| UBC | No |
| UBC9 | No |
| UBD | No |
| UBE2A | No |
| UBE2B | No |
| UBE2N | No |
| UBE2T | No |
| UBE2V1 | No |
| UBE2V2 | No |
| UBE4B | No |
| UBIA1 | No |
| UBIM | No |
| UBP10 | No |
| UBP2 | No |
| UBP28 | No |
| UBP32 | No |
| UBP4 | No |
| UBP47 | No |
| UBP6 | No |
| UBR5 | No |
| UBS3A | No |
| UCHL1 | No |
| UFL1 | No |
| UFO | No |
| UHRF1 | No |
| UHRF2 | No |
| ULA1 | No |
| UNG | No |
| URFB1 | No |
| US6NL | No |
| USE1 | No |
| USP1 | No |
| USP44 | No |
| USP6 | No |
| USP8 | Yes |
| UVSSA | No |
| VATH | No |
| VAV | No |
| VAV1 | No |
| VAV2 | No |
| VAV3 | No |
| VEGFA | No |
| VEGFC | No |
| VGFR1 | No |
| VGFR2 | No |
| VGFR3 | No |
| VHL | Yes |
| VIME | No |
| VMA5A | No |
| VMP1 | No |
| VOPP1 | No |
| VRK1 | No |
| VTI1A | No |
| VWA2 | No |
| WAS | Yes |
| WBP1 | No |
| WDR11 | No |
| WDR48 | No |
| WFDC2 | No |
| WHSC1 | No |
| WHSC1L1 | No |
| WIF1 | Yes |
| WIPI1 | No |
| WIPI2 | No |
| WIPI3 | No |
| WIPI4 | No |
| WISP1 | No |
| WN10B | No |
| WNK1 | No |
| WNK2 | No |
| WNK3 | No |
| WNK4 | No |
| WNT1 | No |
| WNT2B | No |
| WNT3 | No |
| WNT4 | No |
| WNT5A | No |
| WNT6 | No |
| WRN | No |
| WT1 | No |
| WWOX | No |
| WWP1 | No |
| WWTR1 | No |
| XAB2 | No |
| XAF1 | No |
| XBP1 | No |
| XIAP | No |
| XPA | No |
| XPC | No |
| XPF | No |
| XPO1 | Yes |
| XRCC1 | No |
| XRCC2 | No |
| XRCC3 | No |
| XRCC4 | No |
| XRCC5 | No |
| XRCC6 | No |
| XRN1 | No |
| YAF2 | No |
| YAP1 | No |
| YBOX1 | No |
| YES | No |
| YETS4 | No |
| YWHAE | No |
| YYAP1 | No |
| ZBT16 | No |
| ZBT17 | No |
| ZBT7A | No |
| ZBT7C | No |
| ZBTB16 | Yes |
| ZBTB4 | No |
| ZC12D | No |
| ZCCHC8 | No |
| ZDH13 | No |
| ZDH17 | No |
| ZEB1 | No |
| ZFHX3 | Yes |
| ZGPAT | No |
| ZKSC3 | No |
| ZMAT3 | No |
| ZMY10 | No |
| ZMY11 | No |
| ZMYM3 | No |
| ZN185 | No |
| ZN217 | No |
| ZN320 | No |
| ZN350 | No |
| ZN365 | No |
| ZN513 | No |
| ZN521 | No |
| ZN655 | No |
| ZN703 | No |
| ZNF198 | No |
| ZNF278 | No |
| ZNF331 | No |
| ZNF384 | No |
| ZNF429 | No |
| ZNF479 | No |
| ZNF521 | No |
| ZNRF3 | No |
| ZRSR2 | No |
| ZSC32 | No |
| ZW10 | No |
| ZWINT | No |
| ZZEF1 | No |

| **Table S2 – VUS in hereditary cancer related genes** | | | | | | |
| --- | --- | --- | --- | --- | --- | --- |
| **Gene** | **c.** | **p.** | **ClinVar*** | **REVEL** | **M-CAP** | **ID** |
| *AIP* | c.784G>A | p.Asp262Asn | Not found | 0.698 | 0.241892 | 1 |
| *KIF1B* | c.2365G>A | p.Ala789Thr | Not found | 0.382 | 0.146809 | 1 |
| *CEP57* | c.833_834insAGCCAATGTTC | p.Val278fs | Pathogenic | Unavailable | Unavailable | 2 |
| *ERCC6* | c.3704A>G | p.Asp1235Gly | Not found | 0.114 | 0.0327 | 2 |
| *GNAS* | c.1462G>A | p.Ala488Thr | Not found | 0.156 | 0.734804 | 2 |
| *SH2B3* | c.587G>T | p.Arg196Leu | Not found | 0.317 | 0.05925 | 2 |
| *RASAL1* | c.2020G>A | p.Ala674Thr | Not found | 0.11 | 0.191067 | 5 |
| *TOP3A* | c.118C>T | p.Arg40Ter | Pathogenic | 0.091 | Unavailable | 5 |
| *SERPINA1* | c.514G>A | p.Gly172Arg | Not found | 0.47 | 0.030488 | 7 |
| *ALK* | c.361C>T | p.Arg121Trp | Not found | 0.017 | 0.039494 | 8 |
| *SETBP1* | c.3962G>A | p.Arg1321His | Not found | 0.354 | 0.052784 | 8 |
| *EGLN2* | c.235G>A | p.Gly79Ser | Not found | 0.093 | 0.048 | 11 |
| *PRKAR1A* | c.1012T>G | p.Ter338Glu | Not found | Unavailable | Unavailable | 11 |
| *PTCH2* | c.704G>A | p.Arg235Gln | VUS | 0.371 | 0.068779 | 11 |
| *ARMC5* | c.2192C>G | p.Pro731Arg | Not found | 0.063 | 0.034354 | 12 |
| *ATM* | c.6257A>T | p.Tyr2086Phe | VUS | 0.108 | 0.046351 | 12 |
| *ATP4A* | c.2039A>T | p.Gln680Leu | Not found | 0.814 | 0.382493 | 12 |
| *ERCC5* | c.56C>T | p.Pro19Leu | Not found | 0.312 | 0.071367 | 12 |
| *BLM* | c.2237C>T | p.A746Val | VUS | 0.331 | 0.080773 | 13 |
| *AIP* | c.124G>A | p.Val42Met | Conflict | 0.805 | 0.645858 | 14 |
| *TMC8* | c.934G>A | p.Gly312Arg | Not found | 0.285 | 0.040069 | 14 |
| *ANKRD26* | c.4514C>T | p.A1505Val | Not found | 0.207 | 0.029007 | 14 |
| *JAK2* | c.802G>A | p.Gly268Ser | Not found | 0.68 | 0.100005 | 15 |
| *SASH1* | c.2795G>A | p.Arg932Gln | Not found | 0.558 | 0.081689 | 15 |
| *PDGFRB* | c.1223C>G | p.Ser408Cys | Not found | 0.498 | 0.127493 | 16 |
| *ALK* | c.2577G>C | p.Glu859Asp | Conflict | 0.426 | 0.259935 | 17 |
| *GNAS* | c.484A>G | p.Met162Val | Not found | 0.203 | 0.094947 | 17 |
| *LIG4* | c.2467T>C | p.Tyr823His | Not found | 0.377 | 0.051129 | 17 |
| *NSD1* | c.4949A>G | p.Asn1650Ser | Not found | 0.24 | 0.028268 | 17 |
| *RNASEL* | c.770G>C | p.Arg257Thr | Not found | 0.167 | 0.030752 | 17 |
| *TGFBR2* | c.373G>A | p.Glu125Lys | Not found | 0.343 | 0.04892 | 17 |
| *ANKRD26* | c.4246G>C | p.Gly1416Arg | Not found | 0.187 | 0.035906 | 17.1 |
| *ATM* | c.1236G>T | p.Trp412Cys | VUS | 0.494 | 0.496258 | 17.1 |
| *SDHA* | c.1687G>A | p.Val563Met | VUS | 0.484 | 0.289257 | 17.1 |
| *MCM4* | c.526C>T | p.Pro176Ser | Not found | 0.202 | 0.054439 | 19 |
| *RASAL1* | c.1403T>C | p.Phe468Ser | Not found | 0.939 | 0.486553 | 19 |
| *SMARCAD1* | c.1031G>A | p.Arg344His | Not found | 0.241 | 0.035644 | 19 |
| *ACD* | c.1335G>C | p.Gln445His | Not found | 0.059 | 0.025387 | 20 |
| *SH2B3* | c.188G>A | p.Arg63Gln | Not found | 0.237 | 0.083138 | 20 |
| *XPC* | c.2404G>A | p.Gly802Ser | Not found | 0.383 | 0.087328 | 20 |
| *ERCC2* | c.648C>G | p.Asp216Glu | Not found | 0.587 | 0.250278 | 21 |
| *TRIM28* | c.2441C>T | p.Pro814Leu | Not found | 0.208 | 0.063752 | 21 |
| *ATR* | c.2306T>A | p.Leu769Gln | Not found | 0.422 | 0.066746 | 21.1 |
| *POLH* | c.40A>G | p.Met14Val | Not found | 0.848 | 0.147301 | 21.1 |
| *ERCC2* | c.2114A>G | p.Asn705Ser | Not found | 0.26 | 0.036931 | 23 |
| *MLH1* | c.1304T>C | p.Leu435Pro | VUS | 0.917 | 0.473622 | 23 |
| *RNASEL* | c.196G>A | p.Ala66Thr | Not found | 0.714 | 0.321082 | 21.2 |
| *TEX15* | c.647C>T | p.Ser216Phe | Not found | Unavailable | Unavailable | 24 |
| *MRE11A* | c.1475C>A | p.Ala492Asp | Conflict | 0.234 | 0.05513 | 24.1 |
| *TRIM28* | c.1073A>C | p.Asn358Thr | Not found | 0.054 | 0.037188 | 24.1 |
| *WRN* | c.436A>G | p.Lys146Glu | VUS | 0.433 | 0.09425 | 25 |
| *ERCC3* | c.2111C>T | p.Ser704Leu | Not found | 0.228 | 0.102902 | 26 |
| *FAN1* | c.149T>G | p.Met50Arg | Not found | 0.761 | 0.122157 | 26 |
| *PDGFRB* | c.2756G>A | p.Arg919Gln | Not found | 0.434 | 0.084265 | 26.1 |
| *PTCH2* | c.3473C>G | p.Thr1158Ser | VUS | 0.211 | 0.100797 | 27 |
| *TSC2* | c.4072C>G | p.Pro1358Ala | VUS | 0.67 | 0.666093 | 27 |
| *XPC* | c.1001C>A | p.Pro334His | Pathogenic | 0.078 | Unavailable | 28 |
| *POLH* | c.1231A>G | p.Lys411Glu | Probably  Pathogenic | 0.192 | Unavailable | 29 |
| *MSH6* | c.1787T>A | p.Phe596Tyr | VUS | 0.678 | 0.190663 | 30 |
| *MSR1* | c.1105C>T | p.Arg369Trp | Not found | 0.095 | 0.029174 | 30 |
| *PTCH1* | c.2689A>G | p.Ile897Val | VUS | 0.241 | 0.071401 | 30 |
| *RAD54L* | c.604C>T | p.Arg202Cys | Not found | 0.714 | 0.153277 | 32 |
| *RASAL1* | c.904C>T | p.Arg302Cys | Not found | 0.38 | 0.070256 | 33 |
| *SDHA* | c.1676A>G | p.Glu559Gly | VUS | 0.355 | 0.348786 | 17, 11 |
| *POLE* | c.3133G>A | p.Asp1045Asn | Not found | 0.326 | 0.030574 | 26.1, 21 |
| *HNF1B* | c.226G>T | p.Gly76Cys | Conflict | 0.915 | 0.926522 | 25, 27, 8 |
| *PTCH2* | c.3233G>T | p.Gly1078Val | Not found | 0,807 | 0.376 | 34 |
| *RUNX1* | c.238_243delGAGGTG | p.Glu80_Val81del | Not found | Unavailable | Unavailable | 34 |

| **Table S3 – variants in repair genes** | | | | | | |
| --- | --- | --- | --- | --- | --- | --- |
| **Gene** | **c.** | **p.** | **ClinVar*** | **REVEL** | **M-CAP** | **ID** |
| TOP3A | c.118C>T | p.Arg40Ter | Pathogenic | 0.091 | Unavailable | 5 |
| APEX1 | c.433G>A | p.Gly145Ser | Not found | 0.776 | 0.34664 | 6 |
| FANCE | c.31G>A | p.Ala11Thr | VUS | 0.028 | 0.04426 | 7 |
| POLG | c.2354G>A | p.Gly785Asp | VUS | 0.331 | 0.11264 | 8 |
| POLG | c.2207A>G | p.Asn736Ser | Conflict | 0.483 | 0.04363 | 8 |
| POLG | c.2145A>T | p.Gln715His | VUS | 0.292 | 0.06104 | 9 |
| ATM | c.6257A>T | p.Tyr2086Phe | VUS | 0.108 | 0.04635 | 12 |
| ERCC5 | c.56C>T | p.Pro19Leu | Not found | 0.312 | 0.07137 | 12 |
| DNTT | c.1121T>G | p.Leu374Arg | Not found | 0.652 | 0.13089 | 14 |
| RAD1 | c.270A>T | p.Leu90Phe | Not found | 0.386 | 0.02567 | 15 |
| POLL | c.715T>C | p.Trp239Arg | Not found | 0.724 | 0.16304 | 16 |
| ATM | c.1236G>T | p.Trp412Cys | VUS | 0.494 | 0.49626 | 17.1 |
| LIG1 | c.2635C>T | p.Arg879Cys | Not found | 0.786 | 0.099654 | 18 |
| MPG | c.311G>A | p.Arg104Gln | Not found | 0.416 | 0.03431 | 18 |
| XPC | c.2404G>A | p.Gly802Ser | Not found | 0.383 | 0.08733 | 20 |
| ERCC2 | c.648C>G | p.Asp216Glu | Not found | 0.587 | 0.25028 | 21 |
| POLE | c.3133G>A | p.Asp1045Asn | Not found | 0.326 | 0.03057 | 21 |
| POLL | c.958G>A | p.Gly320Arg | Not found | 0.477 | 0.06531 | 21 |
| CHAF1B | c.947G>T | p.Arg316Leu | Not found | 0.644 | 0.22171 | 21.1 |
| POLH | c.40A>G | p.Met14Val | Not found | 0.848 | 0.1473 | 21.1 |
| ERCC2 | c.2114A>G | p.Asn705Ser | Not found | 0.26 | 0.03693 | 23 |
| MLH1 | c.1304T>C | p.Leu435Pro | VUS | 0.917 | 0.47362 | 23 |
| SETMAR | c.532G>C | p.Val178Leu | Not found | 0.386 | 0.10354 | 21.2 |
| DCLRE1B | c.293A>G | p.Asn98Ser | Not found | 0.71 | 0.0266 | 24 |
| WRN | c.436A>G | p.Lys146Glu | VUS | 0.433 | 0.09425 | 25 |
| ERCC3 | c.2111C>T | p.Ser704Leu | Not found | 0.228 | 0.1029 | 26 |
| FAN1 | c.149T>G | p.Met50Arg | Not found | 0.761 | 0.12216 | 26 |
| RBBP8 | c.298C>T | p.Arg100Trp | Pathogenic | 0.753 | 0.05147 | 26 |
| RDM1 | c.52_53del | p.Gln18fs | Not found | Unavailable | Unavailable | 26 |
| MSH5 | c.1738T>G | p.Cys580Gly | Not found | 0.486 | 0.02897 | 27 |
| NEIL2 | c.772C>T | p.Gln258Ter | Not found | Unavailable | Unavailable | 27 |
| FANCI | c.1111A>G | p.Ser371Gly | Conflict | 0.505 | 0.06574 | 29 |
| POLN | c.2509delC | p.Gln837fs | Not found | Unavailable | Unavailable | 29 |
| MSH6 | c.1787T>A | p.Phe596Tyr | VUS | 0.678 | 0.19066 | 30 |
| PARP3 | c.806C>T | p.Pro269Leu | Not found | 0.52 | 0.13159 | 30 |
| PARP3 | c.1462C>T | p.Gln488Ter | Not found | Unavailable | Unavailable | 30 |
| POLG | c.376C>T | p.Arg126Cys | Not found | 0.385 | 0.50517 | 30 |
| TOPBP1 | c.2879C>A | p.Ser960Tyr | Not found | 0.451 | 0.11732 | 30 |
| RAD54L | c.604C>T | p.Arg202Cys | Not found | 0.714 | 0.15328 | 32 |
| RBBP8 | c.1487G>A | p.Arg496Gln | Not found | 0.199 | 0.04174 | 33 |

| **Table S4 – variants in carcinogenisis related genes** | | | | | | |
| --- | --- | --- | --- | --- | --- | --- |
| **Gene** | **c.** | **p.** | **ClinVar*** | **REVEL** | **M-CAP** | **ID** |
| *AIP* | c.124G>A | p.Val42Met | Conflict | 0.805 | 0.645858 | 1 |
| *FGF6* | c.572G>A | p.Arg191Gln | Not found | 0.508 | 0.118585 | 1 |
| *KIF1B* | c.2365G>A | p.Ala789Thr | Not found | 0.382 | 0.146809 | 1 |
| *OBSCN* | c.4531G>T | p.Glu1511Ter | Not found | Unavailable | Unavailable | 1 |
| *RNF139* | c.47A>G | p.Gln16Arg | Not found | 0.035 | 0.027986 | 1 |
| *CEP57* | c.833_834ins | p.Val278fs | Pathogenic | Unavailable | Unavailable | 2 |
| *BAD* | c.221G>A | p.Ser74Asn | Not found | 0.209 | 0.048689 | 3 |
| *MTOR* | c.2150G>A | p.Arg717Gln | Not found | 0.438 | 0.20482 | 3 |
| *NAV2* | c.2336A>G | p.Asn779Ser | Not found | 0.382 | 0.154017 | 3 |
| *CACNA1D* | c.3185A>G | p.Tyr1062Cys | Not found | 0.948 | 0.372564 | 4 |
| *CBLC* | c.200G>A | p.Gly67Glu | Not found | 0.265 | 0.219271 | 4 |
| *LEF1* | c.1153G>A | p.Ala385Thr | Not found | 0.49 | 0.358028 | 4 |
| *PRPF40B* | c.413C>T | p.Ser138Leu | Not found | 0.797 | 0.099105 | 4 |
| *COL1A1* | c.2467C>G | p.Pro823Ala | VUS | 0.706 | 0.37647 | 5 |
| *MIB2* | c.1181A>G | p.Lys394Arg | Not found | 0.185 | 0.148308 | 5 |
| *NRG1* | c.298C>A | p.Leu100Ile | Not found | 0.316 | 0.072291 | 5 |
| *RTN4* | c.230G>C | p.Gly77Ala | Not found | 0.55 | 0.048678 | 5 |
| *TOP3A* | c.118C>T | p.Arg40Ter | Pathogenic | 0.091 | Unavailable | 5 |
| *PAK7* | c.1652C>T | p.Ser551Leu | Not found | 0.531 | 0.066542 | 6 |
| *SFRP4* | c.344A>G | p.Tyr115Cys | Not found | 0.897 | 0.205348 | 6 |
| *SLC45A3* | c.1456C>T | p.Arg486Trp | Not found | 0.452 | 0.054864 | 6 |
| *FANCE* | c.31G>A | p.Ala11Thr | VUS | 0.028 | 0.044257 | 7 |
| *MIB2* | c.1824_1830del | p.Asn608fs | Not found | Unavailable | Unavailable | 7 |
| *SPEN* | c.1475C>G | p.Ala492Gly | Not found | 0.543 | 0.026191 | 7 |
| *TET2* | c.3434G>T | p.Gly1145Val | Not found | 0.522 | 0.045371 | 7 |
| *ALDH2* | c.343G>A | p.Asp115Asn | Not found | 0.365 | 0.060168 | 8 |
| *CACNA1D* | c.3557G>A | p.Arg1186Gln | Not found | 0.782 | 0.223695 | 8 |
| *EGLN1* | c.454C>G | p.Arg152Gly | Not found | 0.234 | 0.759045 | 8 |
| *KMT2D* | c.10193T>A | p.Met3398Lys | Not found | 0.449 | 0.452326 | 8 |
| *NUTM2B* | c.1093C>T | p.Arg365Ter | Not found | Unavailable | Unavailable | 8 |
| *POLG* | c.2354G>A | p.Gly785Asp | VUS | 0.331 | 0.112636 | 8 |
| *POLG* | c.2207A>G | p.Asn736Ser | Conflict | 0.483 | 0.043629 | 8 |
| *TGFB1* | c.1022C>T | p.Ala341Val | Not found | 0.444 | 0.207902 | 8 |
| *ECT2* | c.1072C>T | p.Gln358Ter | Not found | Unavailable | Unavailable | 9 |
| *GOLGA5* | c.1120G>A | p.Glu374Lys | Not found | 0.392 | 0.034612 | 9 |
| *MBD4* | c.939delA | p.Lys313fs | Not found | Unavailable | Unavailable | 9 |
| *POLG* | c.2145A>T | p.Gln715His | VUS | 0.292 | 0.061035 | 9 |
| *TTK* | c.325T>C | p.Phe109Leu | Not found | 0.502 | 0.069219 | 9 |
| *ZFHX3* | c.625C>T | p.Arg209Trp | Not found | 0.226 | 0.044526 | 9 |
| *KDSR* | c.293A>G | p.Y98Cys | Not found | 0.404 | 0.113163 | 10 |
| *ARHGEF10* | c.3379C>T | p.Arg1127Cys | Not found | 0.479 | 0.23691 | 11 |
| *FER* | c.1286A>T | p.Asp429Val | Not found | 0.539 | 0.075194 | 11 |
| *PRKAR1A* | c.1012T>G | p.Ter338Glu | Not found | Unavailable | Unavailable | 11 |
| *PTCH2* | c.704G>A | p.Arg235Gln | VUS | 0.371 | 0.068779 | 11 |
| *SDHA* | c.1687G>A | p.Val563Met | VUS | 0.484 | 0.289257 | 11 |
| *THADA* | c.2011C>G | p.Leu671Val | Not found | 0.294 | 0.066494 | 11 |
| *ATM* | c.6257A>T | p.Tyr2086Phe | VUS | 0.108 | 0.046351 | 12 |
| *ATP4A* | c.2275A>G | p.Met759Val | Not found | 0.861 | 0.217854 | 12 |
| *ERCC5* | c.56C>T | p.Pro19Leu | Not found | 0.312 | 0.071367 | 12 |
| *CBLB* | c.2726G>A | p.Arg909Q | Not found | 0.296 | 0.055295 | 12 |
| *NAA15* | c.850T>G | p.W284Gly | Not found | 0.221 | 0.057078 | 12 |
| *HOXD11* | c.902G>A | p.Arg301Gln | Not found | 0.582 | 0.14319 | 12 |
| *KMT2D* | c.181G>A | p.Gly61Ser | Not found | 0.315 | 0.185873 | 12 |
| *MIB2* | c.2509C>G | p.Gln837Glu | Not found | 0.069 | 0.461417 | 12 |
| *ATP4A* | c.2039A>T | p.Gln680Leu | Not found | 0.814 | 0.382493 | 13 |
| *DNMT3B* | c.73G>A | p.Gly25Arg | VUS | 0.477 | 0.219053 | 13 |
| *FLCN* | c.303G>C | p.Glu101Asp | VUS | 0.557 | 0.111147 | 13 |
| *TNIP2* | c.121C>T | p.Arg41Cys | Not found | 0.168 | 0.45214 | 13 |
| *WNK1* | c.2786C>T | p.Ser929Leu | VUS | 0.287 | 0.028356 | 13 |
| *AFF1* | c.647C>G | p.Ser216Cys | Not found | 0.313 | 0.154573 | 14 |
| *AIP* | c.784G>A | p.Asp262Asn | Not found | 0.698 | 0.241892 | 14 |
| *DNMT3B* | c.274C>T | p.Arg92Trp | Not found | 0.528 | 0.148804 | 14 |
| *DNTT* | c.1121T>G | p.Leu374Arg | Not found | 0.652 | 0.130888 | 14 |
| *FRAT1* | c.214C>T | p.Arg72Trp | Not found | 0.168 | 0.464638 | 14 |
| *LMNA* | c.692G>A | p.Arg231Gln | VUS | 0.492 | 0.079215 | 14 |
| *OBSCN* | c.1513G>A | p.Val505Met | Not found | 0.373 | 0.120659 | 14 |
| *ZNF687* | c.3344G>A | p.Arg1115Gln | Not found | 0.12 | 0.121482 | 14 |
| *CHCHD7* | c.206C>T | p.Thr69Met | Not found | 0.217 | 0.064883 | 15 |
| *FOXO1* | c.311G>C | p.Gly104Ala | Not found | 0.282 | 0.913861 | 15 |
| *NFKB2* | c.1972C>T | p.Arg658Trp | Not found | 0.187 | 0.131075 | 15 |
| *SASH1* | c.2795G>A | p.Arg932Gln | Not found | 0.558 | 0.081689 | 15 |
| *TYK2* | c.1324C>T | p.Arg442Trp | Not found | 0.315 | 0.223598 | 15 |
| *CTBP1* | c.1075G>A | p.Ala359Thr | Not found | 0.197 | 0.102204 | 16 |
| *MYEOV* | c.633delG | p.Leu211fs | Not found | Unavailable | Unavailable | 16 |
| *NAV2* | c.2308C>T | p.Arg770Trp | VUS | 0.309 | 0.136484 | 16 |
| *TTK* | c.553C>T | p.Arg185Trp | Not found | 0.304 | 0.199525 | 16 |
| *KMT2E* | c.1652A>T | p.Glu551Val | Not found | 0.625 | 0.329841 | 16 |
|  |  |  |  |  |  |  |
| *CDH23* | c.361C>T | p.Arg121Trp | VUS | 0.583 | 0.062861 | 17 |
| *CTNND2* | c.587C>T | p.Pro196Leu | Not found | 0.675 | 0.225518 | 17 |
| *DKK4* | c.488C>T | p.Thr163Met | Not found | 0.466 | 0.076299 | 17 |
| *EHBP1* | c.1099C>T | p.Pro367Ser | Not found | 0.295 | 0.055892 | 17 |
| *HSP90AA1* | c.836A>T | p.Lys279Met | Not found | 0.321 | 0.037503 | 17 |
| *TBP* | c.171_183del | p.Gln57fs | Not found | Unavailable | Unavailable | 17 |
| *TGFBR2* | c.373G>A | p.Glu125Lys | Not found | 0.343 | 0.04892 | 17 |
| *TMF1* | c.716G>A | p.Ser239Asn | Not found | 0.28 | 0.026406 | 17 |
| *WNK4* | c.2545C>T | p.Arg849Cys | VUS | 0.767 | 0.506628 | 17 |
| *WNT2B* | c.49C>T | p.Arg17Cys | Not found | 0.386 | 0.521818 | 17 |
| *KAT6B* | c.933G>A | p.Met311Ile | VUS | 0.442 | 0.036755 | 17 |
| *NMI* | c.919G>A | p.Glu307K | Not found | 0.154 | 0.041432 | 17 |
| *FOXO3* | c.79C>G | p.Arg27Gly | Not found | 0.647 | 0.965442 | 17.1 |
| *ATM* | c.1236G>T | p.Trp412Cys | VUS | 0.494 | 0.496258 | 17.1 |
| *TBK1* | c.231_232insTTTTTTT | p.T77Phes | Not found | Unavailable | Unavailable | 17.1 |
| *TBK1* | c.233C>T | p.T78Ile | Not found | 0.136 | 0.086118 | 17.1 |
| *TNIP3* | c.78A>T | p.Glu26Asp | Not found | 0.059 | 0.057249 | 17.1 |
| *ERBB2* | c.2689C>T | p.Arg897Trp | Not found | 0.748 | 0.229027 | 18 |
| *IRF4* | c.1019C>T | p.Ala340Val | Not found | 0.52 | 0.084032 | 18 |
| *KMT2A* | c.5065C>T | p.Pro1689Ser | Not found | 0.313 | 0.092937 | 17.1 |
| *MPG* | c.311G>A | p.Arg104Gln | Not found | 0.416 | 0.034308 | 18 |
| *NBAS* | c.1669T>C | p.Tyr557His | Not found | 0.605 | 0.054407 | 17.1 |
| *SHC3* | c.1489G>A | p.Glu497Lys | Not found | 0.252 | 0.065497 | 18 |
| *MKL2* | c.1570G>C | p.Asp524His | Not found | 0.438 | 0.065941 | 19 |
| *PTPN13* | c.3580G>A | p.Gly1194Ser | Not found | 0.598 | 0.039702 | 19 |
| *PTPN13* | c.3581G>T | p.Gly1194Val | Not found | 0.64 | 0.061049 | 19 |
| *RASAL1* | c.1403T>C | p.Phe468Ser | Not found | 0.939 | 0.486553 | 19 |
| *MINA* | c.602C>T | p.Pro201Leu | Not found | 0.48 | 0.060029 | 19 |
| *WNK1* | c.4949G>C | p.Ser1650Thr | Not found | 0.385 | 0.068688 | 19 |
| *EFS* | c.155T>A | p.Leu52Ter | Not found | Unavailable | Unavailable | 20 |
| *FLT3* | c.580G>A | p.Val194Met | Not found | 0.219 | 0.029824 | 20 |
| *HEY1* | c.218C>T | p.Ser73Phe | Not found | 0.423 | 0.075209 | 20 |
| *MPP3* | c.617C>A | p.Ser206Tyr | Not found | 0.575 | 0.071302 | 20 |
| *XPC* | c.2404G>A | p.Gly802Ser | Not found | 0.383 | 0.087328 | 20 |
| *AFF3* | c.2116_2117del | p.Leu706fs | Not found | Unavailable | Unavailable | 21 |
| *AFF3* | c.2112_2113insAG | p.Asp705fs | Not found | Unavailable | Unavailable | 21 |
| *ERCC2* | c.648C>G | p.Asp216Glu | Not found | 0.587 | 0.250278 | 21 |
| *PABPC1* | c.1240C>T | p.Pro414Ser | Not found | 0.683 | 0.025635 | 21 |
| *POLE* | c.3133G>A | p.Asp1045Asn | Not found | 0.326 | 0.030574 | 21 |
| *POLL* | c.133G>A | p.Gly45Arg | Not found | 0.477 | 0.065309 | 21 |
| *FAT4* | c.5993G>A | p.Gly1998Asp | Not found | 0.861 | 0.761234 | 21 |
| *NUTM2A* | c.2280delC | p.Ser760Phes | Not found | Unavailable | Unavailable | 21 |
| *CASR* | c.74G>A | p.Arg25Gln | VUS | 0.542 | 0.132106 | 22 |
| *CHAF1B* | c.947G>T | p.Arg316Leu | Not found | 0.644 | 0.221705 | 21.1 |
| *MIB2* | c.2015G>A | p.Arg672Gln | Not found | 0.32 | 0.090215 | 22 |
| *POLH* | c.40A>G | p.Met14Val | Not found | 0.848 | 0.147301 | 21.1 |
| *UBR5* | c.6631delA | p.Ile2211fs | Not found | Unavailable | Unavailable | 21.1 |
| *CIC* | c.4783T>C | p.Ser1595Pro | VUS | 0.293 | 0.353904 | 21.2 |
| *TOP1* | c.1625C>T | p.P542Leu | Not found | 0.372 | 0.03784 | 21, 21.2 |
| *HLTF* | c.2440C>T | p.P814Ser | Not found | 0.277 | 0.117256 | 21, 21.2 |
| *DDR1* | c.124A>G | p.Ile42Val | Not found | 0.205 | 0.717166 | 23 |
| *ERCC2* | c.2114A>G | p.Asn705Ser | Not found | 0.26 | 0.036931 | 23 |
| *FLT1* | c.3602C>T | p.Pro1201Leu | Not found | 0.433 | 0.049838 | 23 |
| *FOXP1* | c.11A>C | p.Glu4Ala | Not found | 0.596 | 0.291848 | 21.2 |
| *JAZF1* | c.685T>C | p.Phe229Leu | Not found | 0.327 | 0.048923 | 23 |
| *MLH1* | c.1304T>C | p.Leu435Pro | VUS | 0.917 | 0.473622 | 23 |
| *RNASEL* | c.196G>A | p.Ala66Thr | Not found | 0.714 | 0.321082 | 21.2 |
| *TYK2* | c.3409C>T | p.Pro1137Ser | Not found | 0.779 | 0.274833 | 21.2 |
| *DCLRE1B* | c.293A>G | p.Asn98Ser | Not found | 0.71 | 0.026601 | 24 |
| *FUBP1* | c.124G>T | p.Ala42Ser | Not found | 0.29 | 0.059871 | 24 |
| *FUBP1* | c.121A>T | p.Ile41Phe | Not found | 0.464 | 0.059998 | 24 |
| *PTPRB* | c.4097G>A | p.Arg1366His | Not found | 0.285 | 0.097712 | 24 |
| *TMF1* | c.928T>A | p.Phe310Ile | Not found | 0.42 | 0.073324 | 24 |
| *ABCB1* | c.3770A>C | p.His1257Pro | Not found | 0.836 | 0.256695 | 24.1 |
| *ABTB1* | c.71T>C | p.Phe24Ser | Not found | 0.913 | 0.474665 | 25 |
| *FAT4* | c.10780C>T | p.Pro3594Ser | Not found | 0.48 | 0.026164 | 25 |
| *IL6ST* | c.898C>T | p.Arg300Cys | Not found | 0.353 | 0.034047 | 24.1 |
| *MRE11A* | c.1475C>A | p.Ala492Asp | Conflict | 0.234 | 0.05513 | 24.1 |
| *PALLD* | c.902T>G | p.Ile301Ser | VUS | 0.314 | 0.066693 | 25 |
| *PRDM2* | c.3027G>C | p.Gln1009His | Not found | 0.349 | 0.033145 | 24.1 |
| *SGK1* | c.313delC | p.Pro105fs | Not found | Unavailable | Unavailable | 24.1 |
| *SGK1* | c.317G>T | p.Ser106Ile | Not found | 0.208 | 0.132277 | 24.1 |
| *WNK1* | c.187A>C | p.Lys63Gln | Not found | 0.214 | 0.893766 | 24.1 |
| *EED* | c.973C>T | p.Arg325Cys | Not found | 0.046 | 0.046729 | 24.1 |
| *POLE* | c.2867A>T | p.Y956Phe | Not found | 0.764 | 0.10924 | 24.1 |
| *WRN* | c.436A>G | p.Lys146Glu | VUS | 0.433 | 0.09425 | 25 |
| *ERCC3* | c.2111C>T | p.Ser704Leu | Not found | 0.228 | 0.102902 | 26 |
| *FLT3* | c.113T>A | p.Ile38Asn | Not found | 0.539 | 0.120434 | 26 |
| *LRP1B* | c.12047C>T | p.Pro4016Leu | Not found | 0.491 | 0.05888 | 26 |
| *RBBP8* | c.298C>T | p.Arg100Trp | Pathogenic | 0.753 | 0.051465 | 26 |
| *RDM1* | c.52_53del | p.Gln18fs | Not found | Unavailable | Unavailable | 26 |
| *SPECC1* | c.2915A>G | p.Gln972Arg | Not found | 0.635 | 0.120956 | 26 |
| *HELQ* | c.1043C>T | p.Ser348Phe | Not found | 0.274 | 0.026296 | 26 |
| *AKAP9* | c.10426A>T | p.Arg3476Ter | Not found | Unavailable | Unavailable | 27 |
| *BCL9* | c.3674G>A | p.Arg1225Gln | Not found | 0.286 | 0.045771 | 26.1 |
| *CEBPA* | c.423C>A | p.His141Gln | VUS | 0.022 | 0.695009 | 27 |
| *DPH1* | c.712C>T | p.Arg238Cys | Not found | 0.537 | 0.146259 | 27 |
| *LEF1* | c.550A>C | p.Met184Leu | Not found | 0.445 | 0.144869 | 26.1 |
| *MSH5* | c.1738T>G | p.Cys580Gly | Not found | 0.486 | 0.028969 | 27 |
| *NEIL2* | c.772C>T | p.Gln258Ter | Not found | Unavailable | Unavailable | 27 |
| *PDGFRB* | c.2756G>A | p.Arg919Gln | Not found | 0.434 | 0.084265 | 26.1 |
| *TSC2* | c.4072C>G | p.Pro1358Ala | VUS | 0.67 | 0.666093 | 27 |
| *WISP1* | c.434C>T | p.Thr145Met | Not found | 0.484 | 0.259198 | 26.1 |
| *CADM3* | c.178C>G | p.Leu60Val | Not found | 0.087 | 0.093218 | 28 |
| *LATS2* | c.2434C>T | p.Leu812Phe | Not found | 0.674 | 0.072322 | 28 |
| *NR4A3* | c.760C>G | p.Leu254Val | Not found | 0.303 | 0.392006 | 28 |
| *RHBDF2* | c.589C>G | p.Arg197Gly | Not found | 0.562 | 0.069398 | 28 |
| *ASPSCR1* | c.934_946del | p.Pro312fs | Not found | Unavailable | Unavailable | 29 |
| *FANCI* | c.1111A>G | p.Ser371Gly | Conflict | 0.505 | 0.065742 | 29 |
| *FOXE1* | c.52G>A | p.Val18Met | Not found | 0.268 | 0.906953 | 29 |
| *PTCH2* | c.3473C>G | p.Thr1158Ser | VUS | 0.211 | 0.100797 | 29 |
| *TRAF7* | c.85A>G | p.Arg29Gly | Not found | 0.188 | 0.030952 | 29 |
| *POLI* | c.941A>G | p.Asp314Gly | Not found | 0.765 | 0.027381 | 29 |
| *ELL* | c.868C>T | p.Arg290Trp | Not found | 0.427 | 0.284902 | 30 |
| *MSH6* | c.1787T>A | p.Phe596Tyr | VUS | 0.678 | 0.190663 | 30 |
| *PARP3* | c.1462C>T | p.Gln488Ter | Not found | Unavailable | Unavailable | 30 |
| *PARP3* | c.806C>T | p.Pro269Leu | Not found | 0.52 | 0.131589 | 30 |
| *POLG* | c.376C>T | p.Arg126Cys | Not found | 0.385 | 0.505167 | 30 |
| *PTCH1* | c.2689A>G | p.Ile897Val | VUS | 0.241 | 0.071401 | 30 |
| *AEN* | c.103C>T | p.Arg35Ter | Not found | Unavailable | Unavailable | 31 |
| *COL3A1* | c.226A>G | p.Asn76Asp | VUS | 0.177 | 0.032859 | 31 |
| *KIF1B* | c.2978A>G | p.Asp993Gly | Not found | 0.171 | 0.035571 | 31 |
| *PRDM2* | c.246_247del | p.Asp82fs | Not found | Unavailable | Unavailable | 31 |
| *PRDM2* | c.249delT | p.Asp83fs | Not found | Unavailable | Unavailable | 31 |
| *TBP* | c.171_192del | p.Gln57fs | Not found | Unavailable | Unavailable | 31 |
| *RAD54L* | c.604C>T | p.Arg202Cys | Not found | 0.714 | 0.153277 | 32 |
| *ABCB1* | c.3322T>C | p.Trp1108Arg | Not found | 0.732 | 0.230591 | 32 |
| *ANO1* | c.2516A>G | p.Asn839Ser | Not found | 0.564 | 0.086185 | 32 |
| *DFFB* | c.205C>G | p.Leu69Val | Not found | 0.372 | 0.053516 | 32 |
| *EFTUD1* | c.1696A>C | p.Ile566Leu | Not found | 0.33 | 0.048597 | 32 |
| *EPN1* | c.1430C>T | p.Pro477Leu | Not found | 0.27 | 0.70404 | 32 |
| *KRT19* | c.980C>T | p.Thr327Met | Not found | 0.672 | 0.165107 | 32 |
| *NBPF3* | c.1591C>T | p.Gln531Ter | Not found | Unavailable | Unavailable | 32 |
| *TYK2* | c.3010G>A | p.Ala1004Thr | Not found | 0.658 | 0.236103 | 32 |
| *ZFHX3* | c.5717C>T | p.Ser1906Leu | Not found | 0.429 | 0.038343 | 32 |
| *FGF4* | c.538G>A | p.Ala180Thr | Not found | 0.716 | 0.20223 | 1, 32 |
| *FGFR4* | c.1202G>C | p.Arg401Pro | Not found | 0.311 | 0.104704 | 1, 32 |
| *LGR6* | c.1339G>A | p.Val447Met | Not found | 0.077 | 0.042737 | 33 |
| *LZTS1* | c.338T>C | p.Leu113Pro | Not found | 0.354 | 0.033885 | 2, 31 |
| *RBBP8* | c.1487G>A | p.Arg496Gln | Not found | 0.199 | 0.041737 | 33 |
| *WNK4* | c. 3653dupT | p.Leu 1218fs | Not found | Unavailable | Unavailable | 33 |
| *ROS1* | c.2539T>C | p.W847Arg | Not found | 0.733 | 0.071616 | 33 |
| *FOXO3* | c.413C>T | p.Pro138Leu | Not found | 0.266 | 0.414753 | 7, 28 |
| *FES* | c.1016C>G | p.Pro339Arg | Not found | 0.44 | 0.311379 | 14, 22 |
| *FHL2* | c.337C>T | p.Arg113Cys | VUS | 0.829 | 0.126992 | 15, 22 |
| *TCF7L2* | c.1427T>A | p.Leu476Gln | Not found | 0.364 | 0.152136 | 20, 26.1 |
| *AIM2* | c.712dupA | p.Thr238fs | Not found | Unavailable | Unavailable | 11, 17, 20 |
| *IFRD2* | *c.1154G>A* | *p.Arg385His* | *Not found* | *0.48* | *0.090624* | *21, 28* |
| *TTK* | *c.323G>C* | *p.Ser108Thr* | *Not found* | *0.403* | *0.085393* | *7, 8, 9, 11, 17* |
| *TBP* | *c.171_177del* | *p.Gln57fs* | *Not found* | *Unavailable* | *Unavailable* | *26, 30* |
| *HNF1B* | *c.226G>T* | *p.Gly76Cys* | *Conflict* | *0.915* | *0.926522* | *8, 25, 27* |
| *CD274* | *c.95C>G* | *p.Pro32Arg* | *Not found* | *0.121* | *0.093327* | *7, 28, 31* |
| ACSL6 | c.690G>T | p.Lys230Asn | Not found | 0.205 | 0.089 | 34 |
| ECT2L | c.2227C>T | p.Gln743Ter | Not provided | Unavailable | Unavailable | 34 |
| ETV1 | c.842G>T | p.Gly281Val | Not found | 0,209 | 0.026 | 34 |
| FURIN | c.1390C>T | p.Arg464Trp | Not found | 0,259 | 0.070 | 34 |
| JAK1 | c.2215G>A | p.Asp739Asn | Not found | 0,757 | 0.206 | 34 |
| KMT2D | c.1759G>A | p.Glu587Lys | Not found | 0,389 | 0.319 | 34 |
| NBAS | c.4741C>A | p.Gln1581Lys | Not found | 0,47 | 0.051 | 34 |
| NEO1 | c.3389G>A | p.Arg1130His | Not found | 0,512 | 0.062 | 34 |
| SCRIB | c.3209C>T | p.Thr1070Met | Not found | 0,534 | 0.398 | 34 |

|  | | **Table S5 - All VUS on splicing sites** | | | | | |
| --- | --- | --- | --- | --- | --- | --- | --- |
| **Gene** | **c.** | | **Gene relation** | **ClinVar*** | **ACMG** | **HSF** | **ID** |
| *ECT2L* | c.1579-1G>C | | Carcinogenisis | NF | VUS | Deleterious | 8 |
| *GSDMA* | c.1021+2T>C | | Carcinogenisis | NF | VUS | Deleterious | 8 |
| *PREX2* | c.2715+2T>C | | Carcinogenisis | NF | VUS | Deleterious | 8 |
| *AFP* | c.1840+2T>C | | Carcinogenisis | NF | VUS | Unavailable | 11 |
| *ATM* | c.1236-1G>T | | Repair and hereditary | VUS | VUS | Deleterious | 17.1 |
| *RBL2* | c.1180-1G>T | | Carcinogenisis | NF | VUS | Deleterious | 17.1 |
| *SMAD4* | Unavailable | | Hereditary | NF | VUS | Unavailable | 19 |
| *TDG* | c.1090+1insAGAGCGTGGAGT | | Repair | NF | VUS | Unavailable | 21.1 |
| *SGK1* | c.271-2insTTTT | | Carcinogenisis | NF | VUS | Unavailable | 24.1 |
| *PTPRE* | c.1524+1G>A | | Carcinogenisis | NF | VUS | Deleterious | 31 |
| *NCOR1* | c.3401-2A>G | | Carcinogenisis | NF | VUS | Deleterious | 33 |
| *RAD51B* | c.1037-1G>A | | Repair | NF | VUS | Deleterious | 5, 28 |

**NF= Not found; Last Clinvar update: October, 5^th^ 2021**

| **Table S6 – variants shared across family members** | | | | | | |
| --- | --- | --- | --- | --- | --- | --- |
| **Gene** | **c.** | **p.** | **ClinVar*** | **REVEL** | **M-CAP** | **Family/ID** |
| *A1CF* | c.826C>T | p.Arg276Ter | Not found | Unavailable | Unavailable | 17 |
| *THADA* | c.65A>C | p.Glu22Ala | Not found | 0.141 | 0.056885 | 17 |
| *COL3A1* | c.560C>T | p.Thr187Ile | Conflict | 0.187 | 0.063677 | 17 |
| *KDR* | c.2312C>T | p.Thr771Met | Not found | 0.478 | 0.105661 | 17 |
| SDHA | c.1676A>G | p.Glu559Gly | VUS | 0.355 | 0.348786 | 17 |
| MSH5 | c.859C>T | p.Arg287Cys | Not found | 0.753 | 0.14541 | 17 |
| *HIC1* | c.1468G>C | p.Gly490Arg | Not found | 0.142 | 0.067907 | 17 |
| *WNK4* | c.3593G>A | p.Arg1198His | Not found | 0.435 | 0.1105 | 17 |
| *NUTM2A* | c.2280delC | p.Ser760fs | Not found | Unavailable | Unavailable | 21 |
| *HLTF* | c.2437C>T | p.Pro813Ser | Not found | 0.277 | 0.117256 | 21 |
| *FAT1* | c.8465T>C | p.Leu2822Pro | Not found | 0.288 | 0.030918 | 21 |
| *MAFK* | c.353C>T | p.Ala118Val | Not found | 0.57 | 0.404094 | 21 |
| *PREX2* | c.61C>T | p.Leu21Phe | Not found | 0.221 | 0.829486 | 21 |
| RAD21 | c.1892T>C | p.Ile631Thr | Not found | 0.382 | 0.08152 | 21 |
| *IFFO1* | c.139G>C | p.Gly47Arg | Not found | 0.462 | 0.983947 | 21 |
| *E2F7* | c.7G>T | p.Val3Leu | Not found | 0.213 | 0.045625 | 24 |
| FAN1 | c.80C>T | p.Ser27Phe | Not found | 0.22 | 0.047098 | 24 |
| *CHAF1B* | c.1478G>A | p.Ser493Asn | Not found | 0.094 | 0.048378 | 24 |
| *ABTB1* | c.490C>T | p.Arg164Ter | Not found | Unavailable | Unavailable | 26 |
| *VEGFC* | c.224C>G | p.Pro75Arg | Not found | 0.545 | 0.030322 | 26 |
| *TBX3* | c.1240G>A | p.Asp414Asn | Not found | 0.31 | 0.620597 | 26 |
| FAN1 | c.149T>G | p.Met50Arg | Not found | 0.761 | 0.122157 | 26 |
| *CDT1* | c.1373C>T | p.Ala458Val | Not found | 0.463 | 0.146513 | 26 |
| *FBLN1* | c.1282G>T | p.Val428Leu | Not found | 0.144 | 0.092556 | 26 |
| *NDRG2* | c.172C>T | p.Arg58Cys | Not found | 0.48 | 0.084001 | 26 |
| *FRMD3* | c.463A>G | p.Ile155Val | Not found | 0.438 | 0.027061 | 26 |
|  |  |  |  |  |  |  |

Figure S1 – Family 17


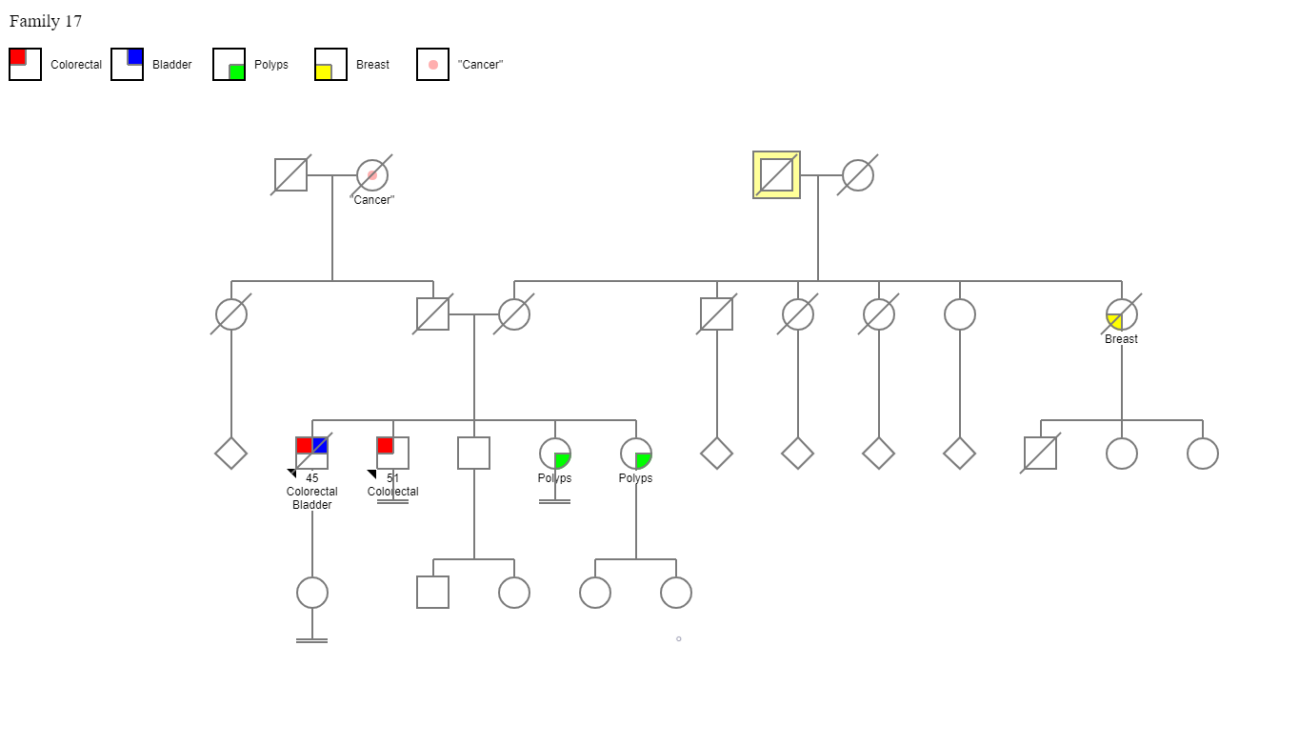


Figure S2 – Family 21


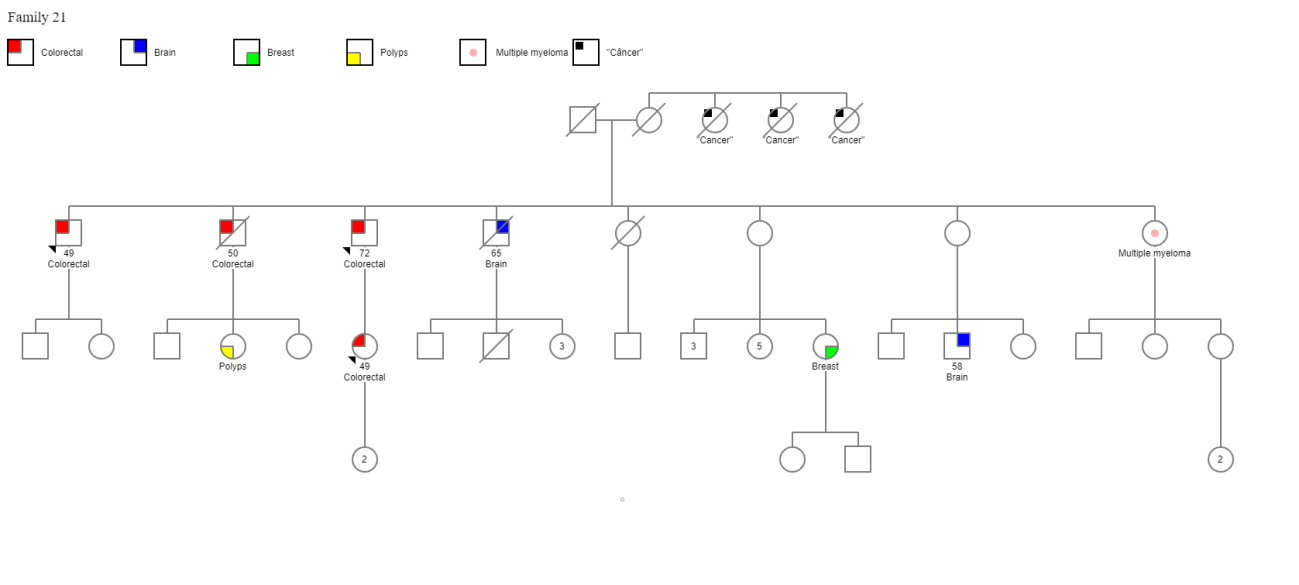


Figure S3 – Family 23

**
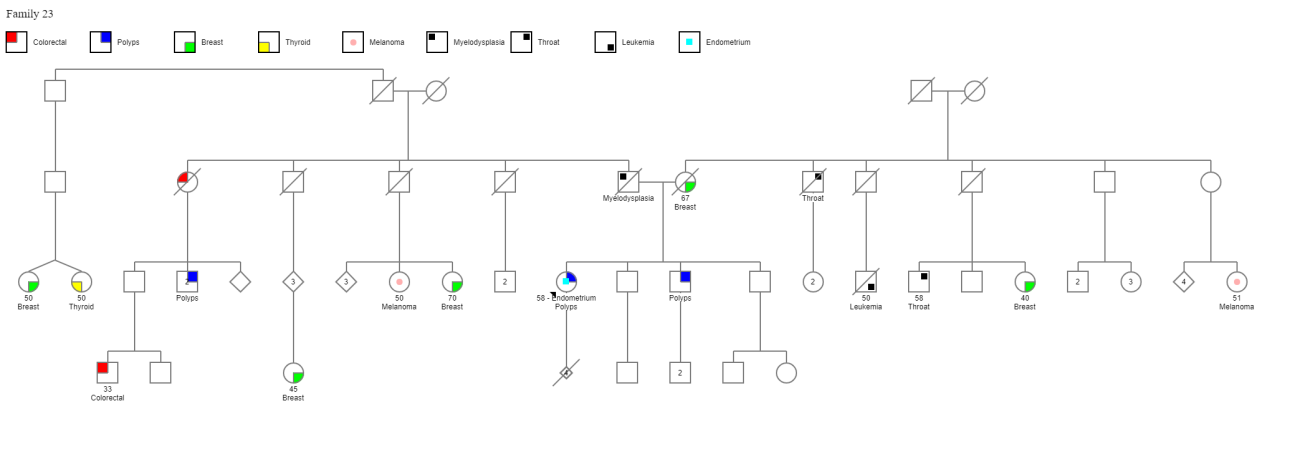
**

Figure S4 – Family 26


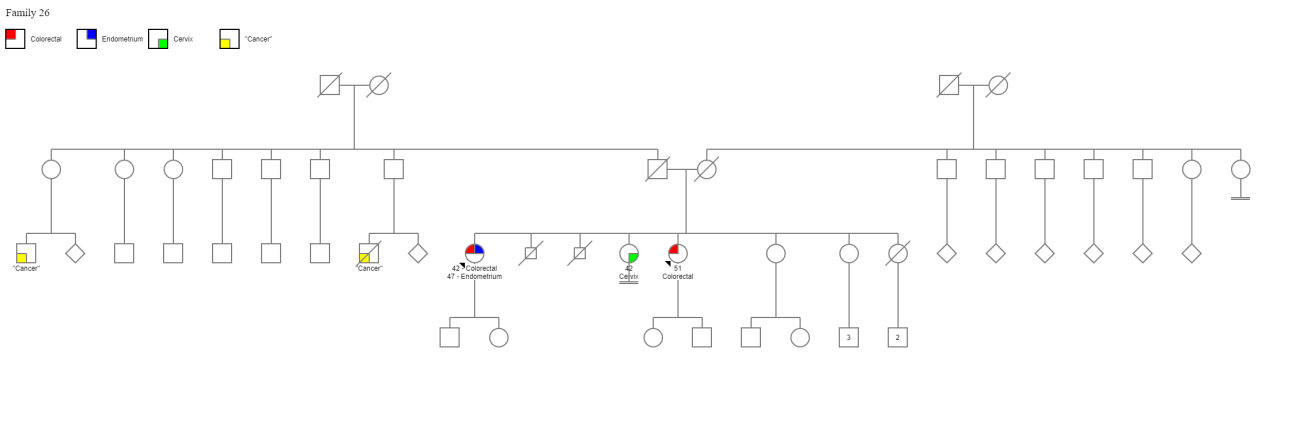


Figure S5 – Family 33


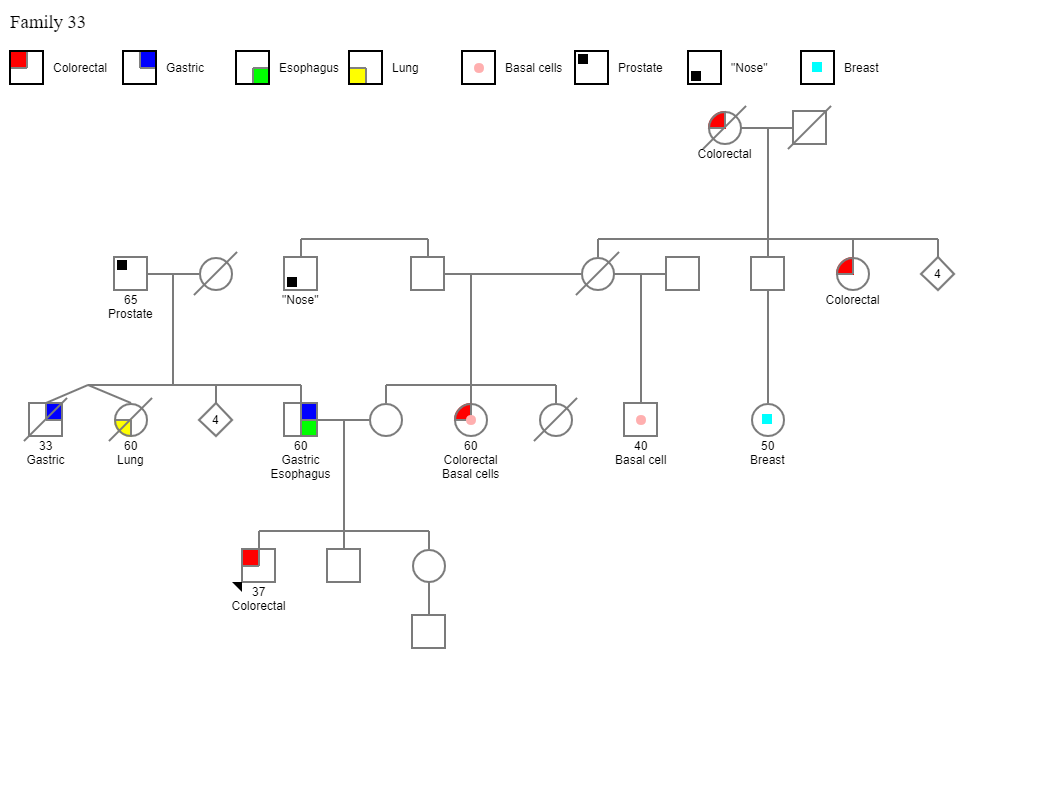


Figure S6 – Family 6


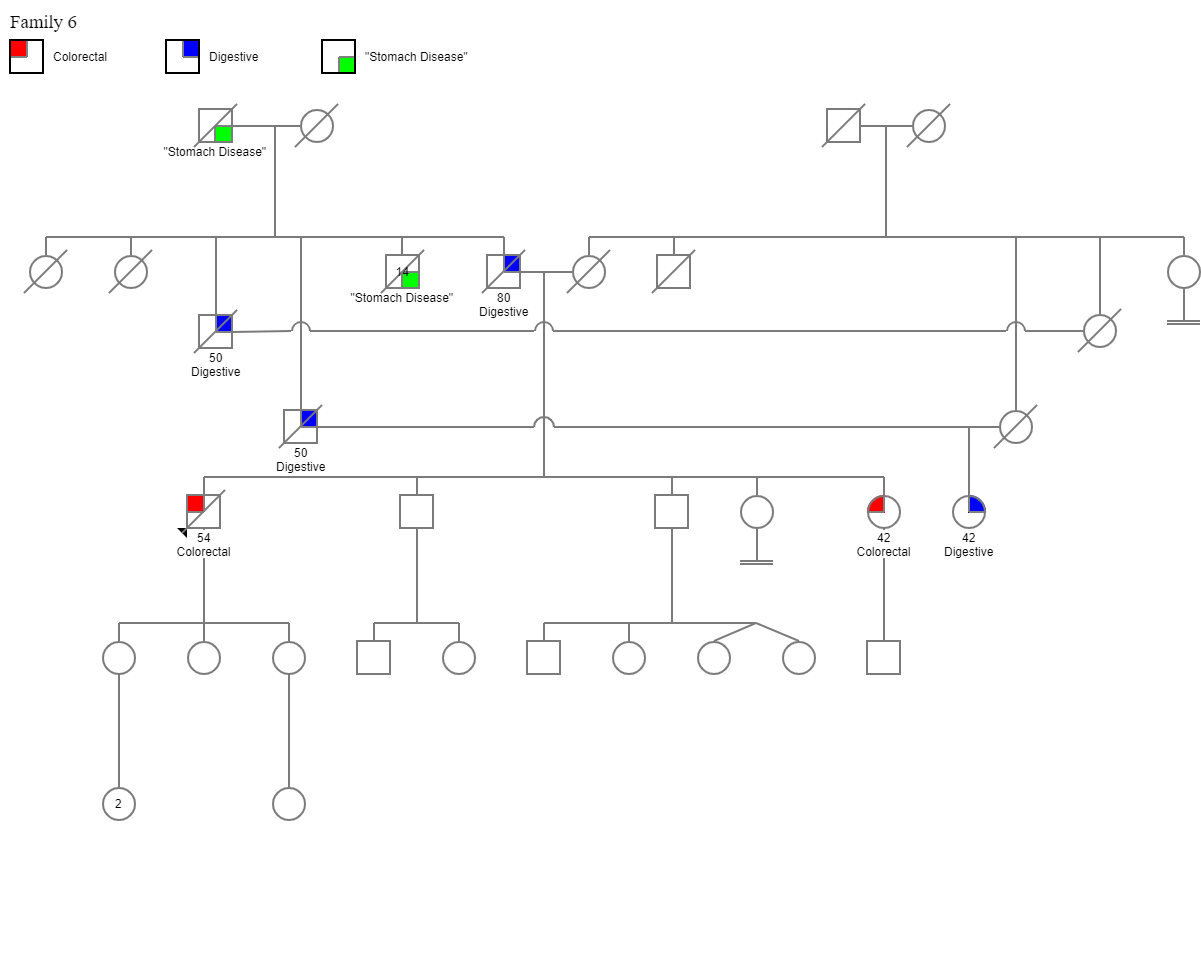


Figure S7 – Family 22


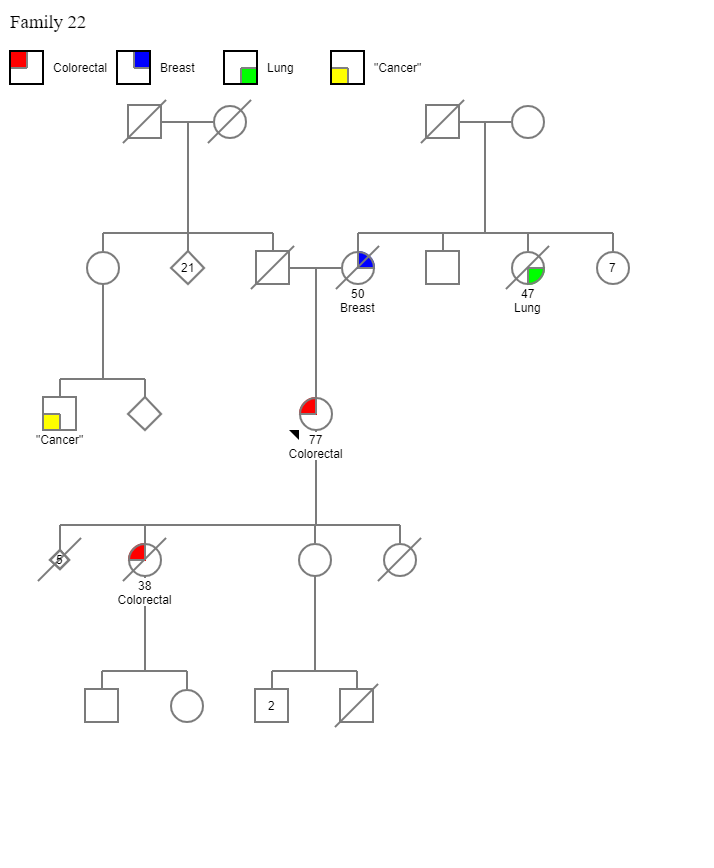


Figure S8 – Family 5


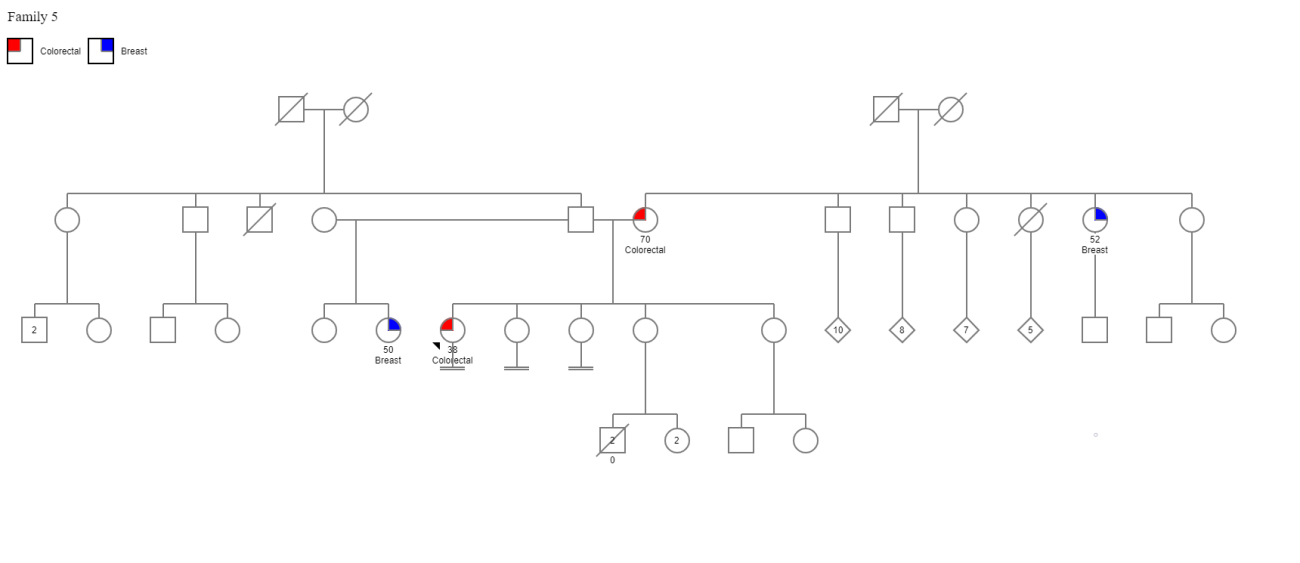


Figure S9 – Figure 3

**
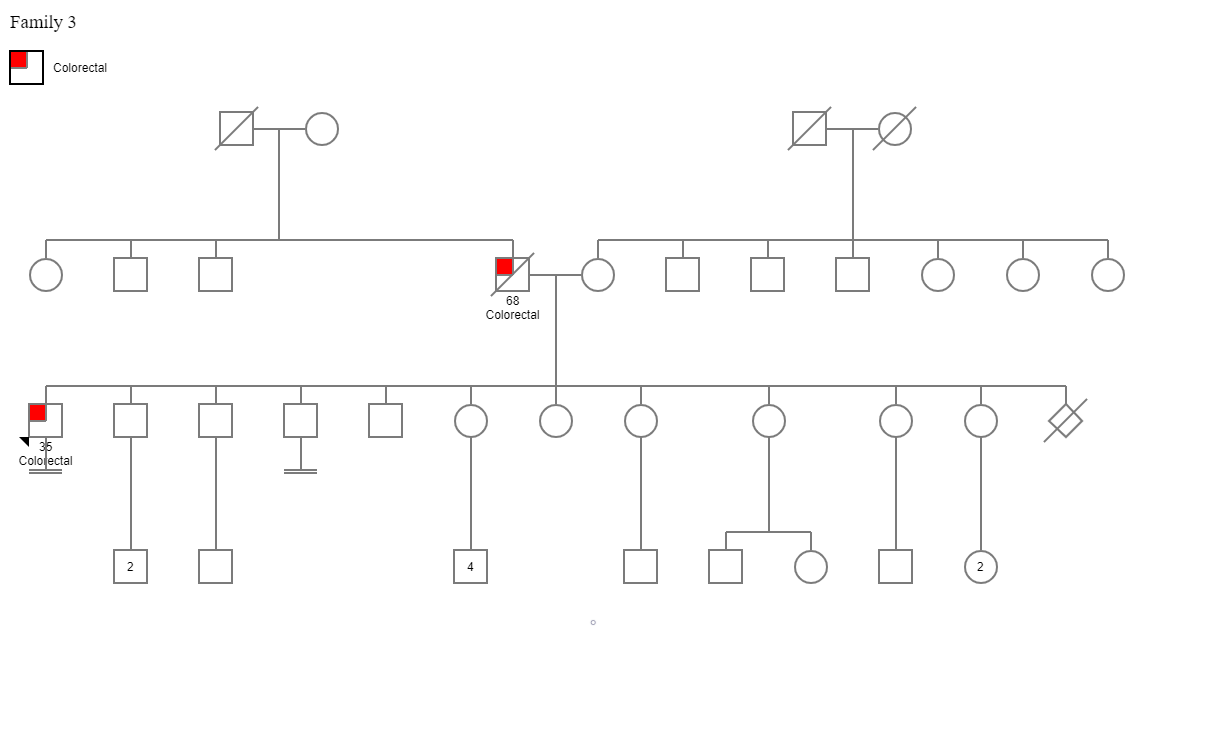
**

Figure S10 – Family 13


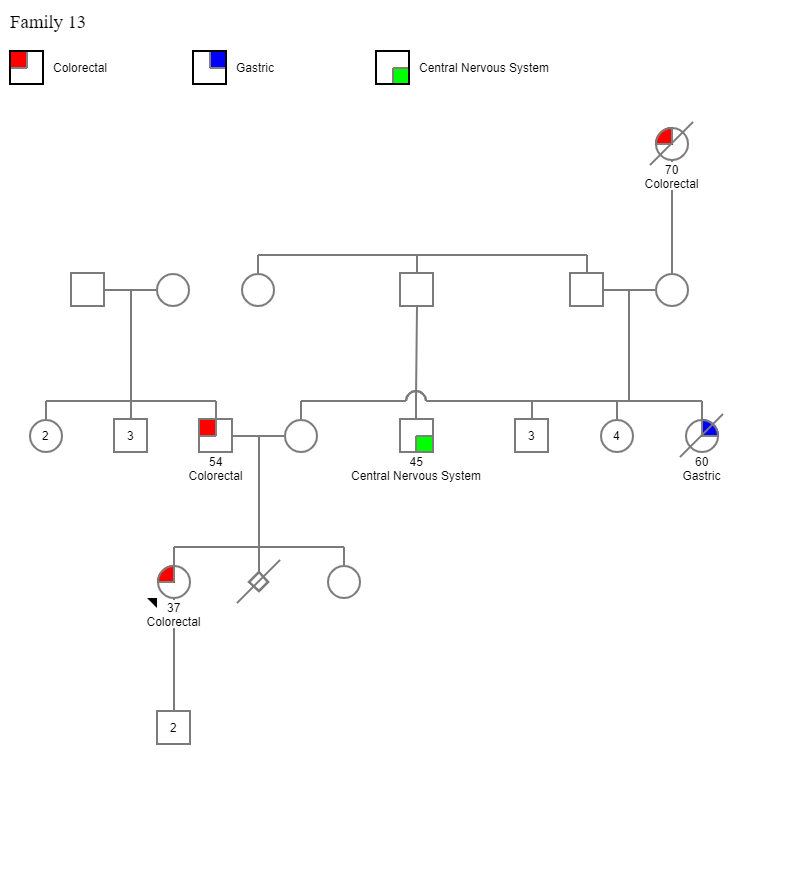

Supplement: Supplementary file 1 — Supplementary Information. [file 41598_2022_6782_MOESM1_ESM.docx]
